# Influenza A virus infection impacts systemic microbiota dynamics and causes quantitative enteric dysbiosis

**DOI:** 10.1186/s40168-017-0386-z

**Published:** 2018-01-10

**Authors:** Soner Yildiz, Béryl Mazel-Sanchez, Matheswaran Kandasamy, Balaji Manicassamy, Mirco Schmolke

**Affiliations:** 1 Department of Microbiology and Molecular Medicine, University Medical Center (CMU), University of Geneva, Rue Michel-Servet 1, CH-1211 Geneva, Switzerland; 2 Department of Microbiology, University of Chicago, Chicago, IL 60637 USA

## Abstract

**Background:**

Microbiota integrity is essential for a growing number of physiological processes. Consequently, disruption of microbiota homeostasis correlates with a variety of pathological states. Importantly, commensal microbiota provide a shield against invading bacterial pathogens, probably by direct competition. The impact of viral infections on host microbiota composition and dynamics is poorly understood. Influenza A viruses (IAV) are common respiratory pathogens causing acute infections. Here, we show dynamic changes in respiratory and intestinal microbiota over the course of a sublethal IAV infection in a mouse model.

**Results:**

Using a combination of 16S rRNA gene-specific next generation sequencing and qPCR as well as culturing of bacterial organ content, we found body site-specific and transient microbiota responses. In the lower respiratory tract, we observed only minor qualitative changes in microbiota composition. No quantitative impact on bacterial colonization after IAV infection was detectable, despite a robust antimicrobial host response and increased sensitivity to bacterial super infection. In contrast, in the intestine, IAV induced robust depletion of bacterial content, disruption of mucus layer integrity, and higher levels of antimicrobial peptides in Paneth cells. As a functional consequence of IAV-mediated microbiota depletion, we demonstrated that the small intestine is rendered more susceptible to bacterial pathogen invasion, in a *Salmonella typhimurium* super infection model.

**Conclusion:**

We show for the first time the consequences of IAV infection for lower respiratory tract and intestinal microbiobiota in a qualitative and quantitative fashion. The discrepancy of relative 16S rRNA gene next-generation sequencing (NGS) and normalized 16S rRNA gene-specific qPCR stresses the importance of combining qualitative and quantitative approaches to correctly analyze composition of organ associated microbial communities. The transiently induced dysbiosis underlines the overall stability of microbial communities to effects of acute infection. However, during a short-time window, specific ecological niches might lose their microbiota shield and remain vulnerable to bacterial invasion.

**Electronic supplementary material:**

The online version of this article (10.1186/s40168-017-0386-z) contains supplementary material, which is available to authorized users.

## Background

The human body hosts a substantial number of symbiotically living microorganisms on basically all surfaces, including the respiratory and the intestinal tract [1, 2]. With the development of culture independent next-generation sequencing (NGS) techniques and the use of axenic animal models, the field of metagenomics research underwent exponential growth. Despite the vast amount of sequencing data, our understanding of the physiological function of microbiota and their dynamics is still a matter of ongoing research. This is especially true with respect to causality under given pathologic conditions. In the intestine, commensal microbes are essential for the breakdown of nutritional products and key producers of essential vitamins [3]. They occupy ecological niches, thus posing a competitive threshold to invasion of pathogenic bacteria [4]. Additionally, commensal microbiota were demonstrated to have an important role in the education of regulatory T cells in the intestinal tract [5]. Consequently, disturbance of microbial balance (dysbiosis) might have substantial consequences, e.g., for host metabolism and adaptive immune responses. These consequences can occur locally and systemically. For instance, both local (respiratory) and distal (gut) microbiota impact lung immune responses [6, 7] against IAV infection. Pathological changes in microbiota composition were extensively studied in a number of chronic (inflammatory) diseases, such as asthma, COPD, cystic fibrosis, or IBD (summarized in [1, 8–12]). Recently, chronic infectious diseases were characterized as a factor, disturbing microbiota balance [13, 14].

Despite these rapid advances, there is little information on the impact of acute viral infection on composition, kinetics, and quantity of commensal microbiota in a given host site. Here, we used a longitudinal approach to study microbiota kinetics after IAV infections and their consequences for the host. Using a combination of 16S rRNA gene NGS, 16S rRNA gene-specific qPCR, and culture of bacterial content, we demonstrate here surprisingly little effect of IAV infection on lower respiratory tract microbiota abundance and composition. In contrast, we find substantial elimination of microbiota from small intestine of IAV-infected mice both by culture-dependent and culture-independent techniques. These changes are transient, independent of the virus subtype and resolve after clearance of viral infection. Despite these differences in microbiota dynamics in the two organ systems, we see consistent IAV-dependent sensitization of lung and intestinal tissue to bacterial super infection.

## Results

### Sublethal respiratory tract IAV infection provokes transient clinical signs

**Fig. 1:**
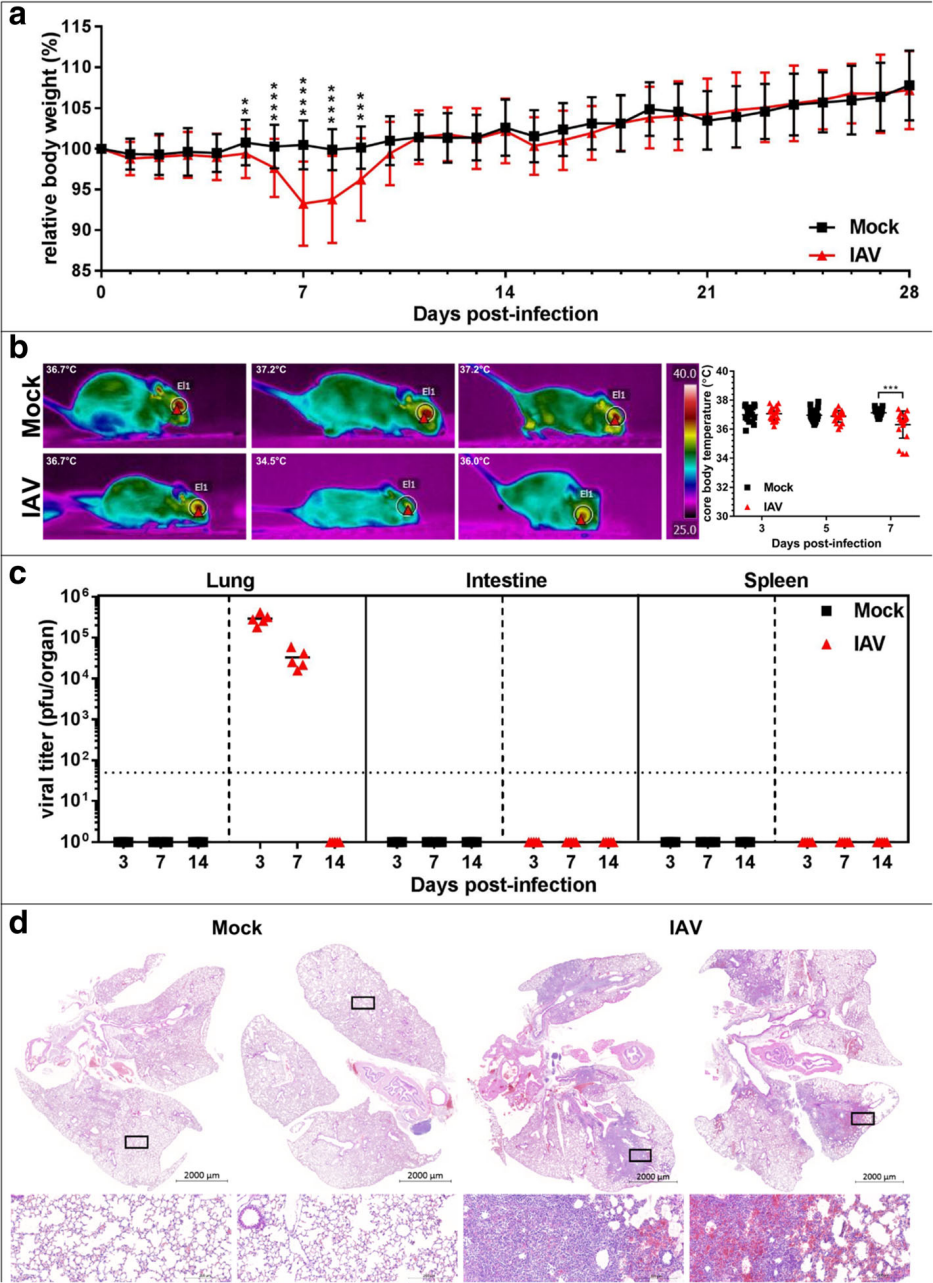
Influenza A virus causes transient pathology in mice**. a** IAV causes transient body weight loss. Average relative initial body weight (%) ± SD is depicted for mock-treated (black line, *n* ≥ 18 per sample time point) and IAV-infected (red line, *n* ≥ 18 per sample time point). Pooled data from two independent experiments are shown. **b** Left side: representative thermal pictures of mock-treated and IAV-infected animals (lower panels), 7 dpi. Right side: quantification of core body temperature on days 3, 5, and 7 post infection, for mock-treated (black squares) and IAV-infected (red triangles) mice, median core temperature is indicated. Pooled data from two independent experiments are shown (*n* = 20 per time point, per group). **c** Individual total organ titers of mock-treated (black squares) and IAV-infected mice (red triangles), median titers (pfu/organ) are indicated. Limit of detection (LoD) 50 pfu is indicated by dotted line (*n* = 5 per time point, per group). **d** H&E stainining of mock-treated (left) and IAV-infected (right) mouse lungs (*n* = 6 per group from two independent experiments). Two representative lungs with indications for magnifications are shown. Lower panels: magnification of healthy vs. inflamed tissue

To better understand dynamics of commensal bacteria in acute infection settings, we decided to track changes in lung and gut microbiota in a longitudinal approach in context of a sublethal viral infection. Most lab-adapted IAV strains are highly lethal in mice and cause substantial body weight loss. Thus, we chose a low pathogenic variant of a H5N1 isolate (A/Viet Nam/1203/2004) [15], causing only mild disease in mice, thus recapitulating transient influenza pathogenesis, as observed in most human patients. Infected adult mice displayed transient and modest weight loss (Fig. 1a, *n* = 18 per time point and experimental group. Note: mean weights are cumulative: until 3 dpi *n* = 90, from 4 and 5 dpi from *n* = 72, from 6 and 7 dpi *n* = 54, from 8–14 dpi *n* = 36, and from 15–28 dpi *n* = 18) with mild sickness behavior. Using thermal imaging, we observed a subtle but significant reduction of core body temperature 7 days post infection (dpi), as indicated by the temperature of the eyes, confirming previous findings [16] (Fig. 1b, *n* = 20 animals per experimental group, representative thermal pictures shown). However, these changes were in average well within the daily body temperature fluctuation of mice [17]. Infected animals recovered weight levels of mock treated animals by 10–11 dpi (Fig 1a). Viral lung clearance was observed between 7 and 14 dpi. Virus replication was restricted to the respiratory tract, as no infectious particles were found in the small intestine or spleen (Fig. 1c, *n* = 5 animals per time point per group). As described before [18], we observed substantial lung infiltrates of mainly mononuclear cells 7 dpi (Fig. 1d, *n* = 6 animals, representative pictures shown).

### IAV causes subtle transient changes in the microbial composition of the lower respiratory tract

Following the symptoms of viral pathogenesis, we sampled total lung (lower respiratory tract (LRT)), proximal small intestine (SI), and feces on 0 (untreated), 3, 5, 7, 14, and 28 dpi or PBS treatment, respectively (*n* = 18 samples per time point and experimental group, samples that did not pass quality controls or analytical thresholds are listed in Additional file 1: Table S1). Composition of microbiota was determined by culture-independent 16S rRNA gene-specific NGS.

**Fig. 2:**
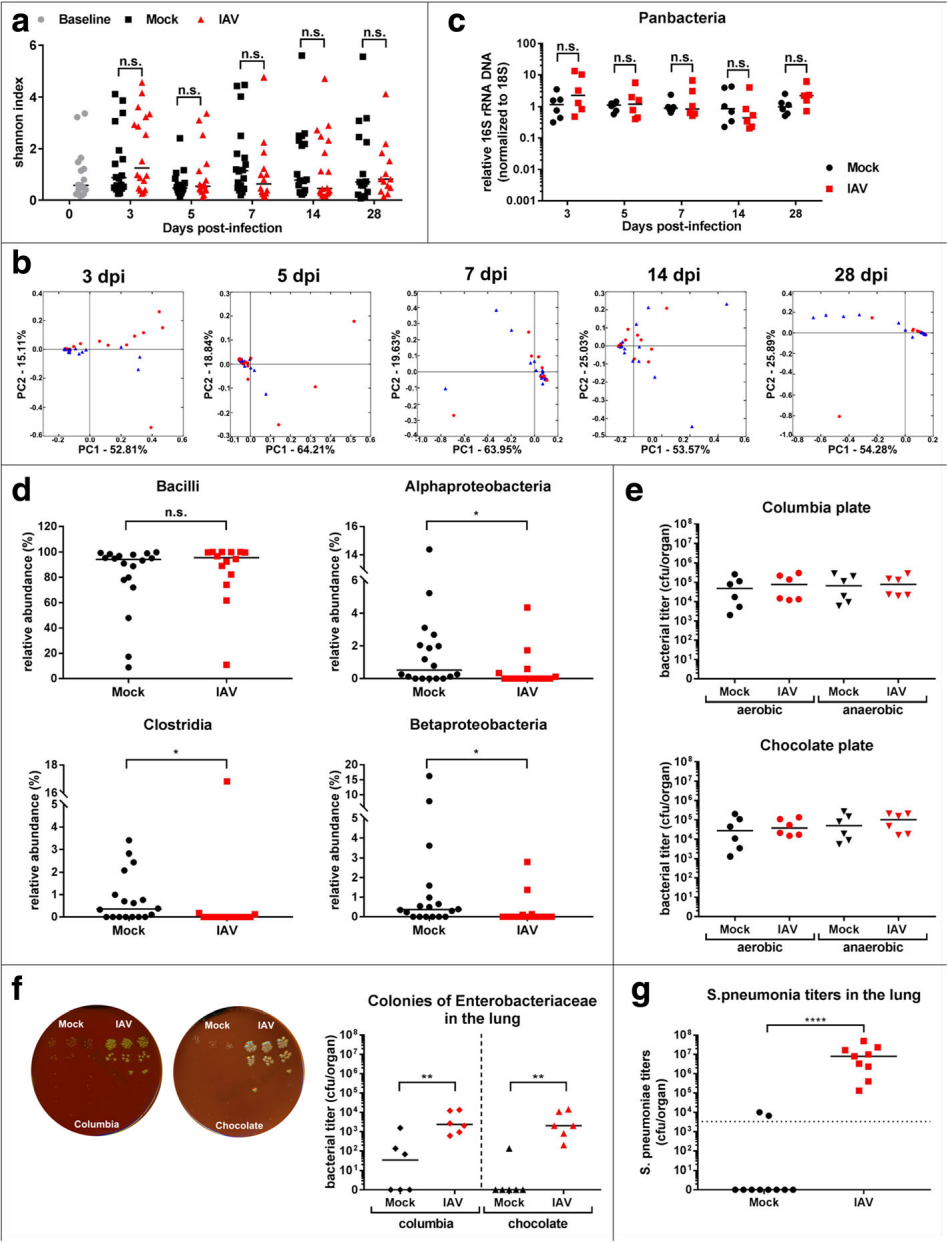
Influenza A virus infection has minor impact on global LRT microbiome. **a** Individual Shannon H-index of LRT microbiota of baseline (gray circles), mock-treated (black squares) and IAV-infected mice (red triangles) pooled from four independent cages are depicted for indicated days of sampling. Median Shannon index per experimental group is indicated. **b** 2D-PCoA plots of LRT microbiota of mock-treated (blue triangle) or IAV-infected mice (red circle) mice at indicated time points post infection. Each symbol represents one individual mouse. Percentages explain variation in PC1 (*x*-axis) and PC2 (*y*-axis). **c** Normalized individual 16S/18S qPCR results (n-fold relative to mean of mock samples) and median are depicted for individual days for mock-treated (black circle) and IAV-infected (red square) (*n* = 18 per time point, per group from two independent experiments, representative data shown). **d** Relative abundance of bacteria of the LRT determined by 16S rRNA gene-specific NGS is indicated for individual classes. Pooled data from two independent experiments with median are shown. **e** Individual bacterial titers (cfu/organ) and median determined under aerobic (left) and anaerobic conditions (right) on Columbia or Chocolate agar are depicted for mock treated (black symbols) and IAV infected (red symbols). Pooled data from two independent experiments are shown (*n* = 6 per group per condition). **f** Left panel: Enterobacteriaceae (OTU-ID 1111294)-like colonies in serial dilutions of LRT homogenate on Columbia (left) and Chocolate agar (right) from mock treated (left half) and infected animals (right half). Representative plates are shown. Right panel: quantification of titers of Escherichia (OTU ID 1111294)-like colonies from mock-treated (black symbols) or IAV-infected LRT homogenates (red symbols). Individual titers (cfu/organ) and median of homogenates grown on indicated agars from two independent experiments are shown (*n* = 6 per group per condition). **g** *S. pneumoniae* bacterial lung titers (cfu/organ) 24 h post infection from mock treated of IAV preinfected animals from two independent experimental sets are shown (mock (*n* = 10), IAV (*n* = 9))

Intuitively, we expected a more robust impact of IAV infection on LRT microbiota, since the local antimicrobial response was expected to be stronger at the site of virus replication and the overall bacterial density is magnitudes lower in the LRT as compared to the SI. To our surprise, we did not detect significant changes in alpha diversity or beta diversity of LRT microbiota throughout the time course of infection (Fig. 2a, b). Analysis of 16S rRNA gene-specific NGS revealed overwhelming presence of *Firmicutes* on phylum level in lung tissue of untreated mice. About 90% of sequences belonged to this phylum; the vast majority was contributed by the class of *Bacilli* (Additional file 2: Figure S1C, for baseline mice). Additional file 3: Figure S2B lists the most abundant OTUs on genus level in untreated (baseline), mock-treated and IAV-infected lungs at the point of most pronounced clinical signs (7 dpi). For all experimental groups, the genus of *Lactobacillus* dominates the LRT microbiome (Additional file 3: Figure S2B). To rule out environmental contamination, we compared the total read counts (Additional file 4: Table S2), taxonomic composition of rarified data, and beta diversity of rarified data of baseline animal organ samples (*n* = 17 for lung, *n* = 18 for intestine) vs. blanks (*n* = 6) or baseline fecal samples (*n* = 18) vs. blanks (*n* = 5), respectively (Additional file 2: Figure S1 A–C and Additional file 5: Table S3). Two sets of blanks (blank A for organ samples, blank B for fecal samples) were required since organ and fecal samples were isolated with different DNA isolation kits, thus might contain potentially different contaminants. Overall read counts for blanks (A and B) were low compared to respective organ or fecal samples (Additional file 4: Table S2). Moreover, taxonomic composition of blanks differed substantially from organ samples. This is of special importance for LRT samples, due to the low abundance microbiota in this body site. Four out of six blank controls contained either no or extremely low count contamination with predominant OTUs found in LRT samples and show high number of *Proteobacteria* (Additional file 5: Table S3). Consequently, PCoA analysis showed separation of the majority of organ samples from blanks (Additional file 2: Figure S1B). Since two blank samples showed higher abundance of *Bacilli* by 16S rRNA gene sequencing (potentially by cross-well contamination during library construction PCR), we cannot fully rule out a minor impact of environmental/experimental contaminants on the here described taxonomic composition of LRT microbiota. In order to rule out false assignment of LRT-derived reads to the genus of *Lactobacillus*, we additionally acquired raw reads from paired, barcode-extracted sequences of each LRT baseline sample. From these, we subsampled 200 reads per sample and blasted them against the NCBI database (nucleotide collection “nr/nt”). Again, the top hit for each sample aligned with 100% sequence identity to the genus *Lactobacillus* in 16/17 samples (data not shown).

Direct comparison of mock-treated and IAV-infected animals 7 dpi revealed subtle but significant reduction of *alpha-* and *beta-proteobacteria* as well as *Clostridia* on class level (Fig. 2d, mock (*n* = 18), IAV (*n* = 14), pooled). These changes were transient and resolved by 14 dpi (data not shown). We next assessed quantitative changes in LRT microbiota by normalizing pan-16S or phylum-specific qPCR values to host 18S DNA-specific qPCR (16S/18S). We observed no significant changes in total microbial DNA or *Firmicutes* DNA abundance in the lung (Fig. 2c, Additional file 3: Figure S2C mock (*n* = 18), IAV (*n* = 18), representative data are shown). By qPCR, we did not detect DNA of *Bacteroidetes* (data not shown), confirming their low abundance in NGS results. These qPCR data were confirmed with lung samples of mice infected with a human H1N1 virus A/Netherlands/602/2009 (Additional file 3: Figure S2D and E). Since 16S rRNA gene-specific qPCR and NGS only reflect the amount of bacterial DNA in a given sample site, we decided to verify these data by plating LRT homogenates on rich agar media and determine the number of cultivatable bacteria in mock-treated and IAV-infected animals (Fig. 2e, mock (*n* = 6), IAV (*n* = 6), pooled). In infected animals, we did not detect an increase in total cultivatable bacteria of the upper respiratory tract, in contrast to a previous report [19] (Additional file 3: Figure S2F). We further found no alterations in the amount of cultivatable bacteria in the LRT, neither under aerobic nor under anaerobic conditions (Fig. 2e). However, we observed increased presence of clear, large colonies within the plated LRT homogenates of IAV-infected animals (Fig. 2f). 16S rRNA gene-specific Sanger sequencing of individual colonies revealed presence of *Enterobacteriaceae* (OTU ID 1111294) in all tested colonies (Additional file 3: Figure S2G, *n* = 6). In order to test the origin of this OTU, we compared its presence in LRT, SI, and fecal samples. OTU 1111294 is found in the lung, in SI, and fecal samples (Additional file 6: Table S4). To support the presence of *Lactobacillus* (see Additional file 3: Figure S2B) in the lung of control mice, we cultured total homogenates of lungs from three animals on Columbia agar, isolated genomic DNA from three randomly chosen colonies and sequenced for 16S rRNA gene segment following colony PCR (see “Methods” section). All sequences confirmed the presence of a member of the *Lactobacillaceae* family (Additional file 3: Figure S2H). Of note, none of the blank samples showed bacterial growth (data not shown).

Previous studies suggested that alteration of growth conditions for commensal and pathogenic bacteria might contribute to establishment of bacterial super infection in the respiratory tract after IAV infection [19, 20]. To test, if super infection is favored under the chosen infection conditions, with little change on LRT microbiota, we used the well-established *S. pneumoniae* super infection model [21]. As previously reported, we could show that sublethal IAV infection indeed increases bacterial LRT titers of *S. pneumoniae* (Fig. 2g, *n* = 10 for mock, *n* = 9 for IAV infected).

Our data show that the LRT microbiota is surprisingly stable against IAV infection and responds only with subtle changes in composition, but without alteration of bacterial abundance, community richness or beta diversity. Nevertheless, growth conditions for a yet to be characterized group of bacteria are enhanced under these conditions.

### IAV provokes quantitative depletion of small intestine microbiota and destruction of mucus layer integrity

We speculated that systemic host immune reactions to the virus might impact microbiota dynamics in distal body sites. In accordance with this idea, recent studies showed an impact of IAV infection on composition of fecal microbiota in mice and patients [22, 23]. The SI microbiota is substantially different and less rich than the fecal microbiota [24] (Additional file 2: Figure S1), and it plays an important role in priming adaptive immune responses [5, 7, 25]. We thus decided to characterize the impact of IAV infection on these particular microbiota.

**Fig. 3:**
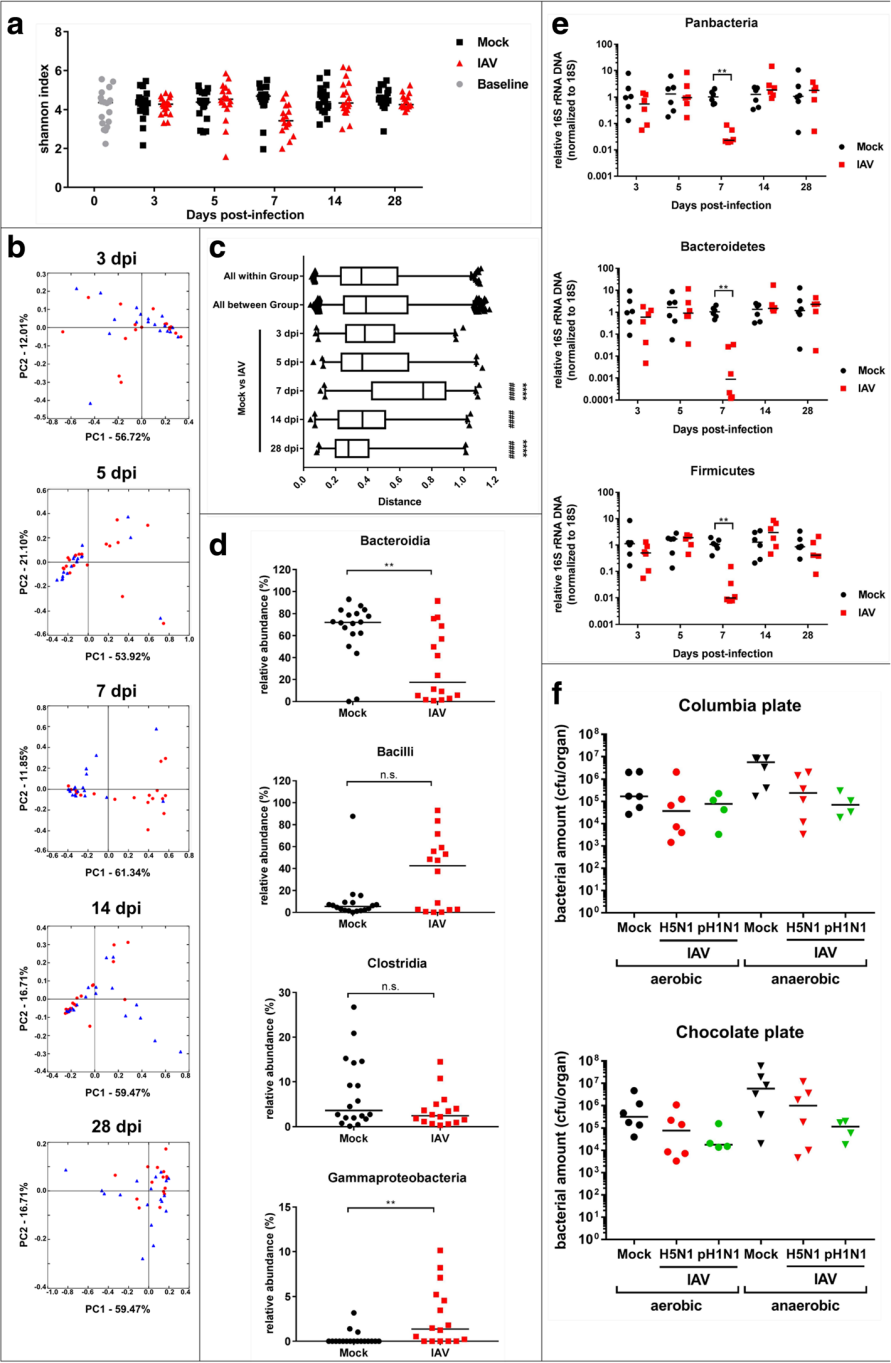
Influenza A virus infection eliminates small intestinal microbiota. **a** Individual Shannon H-index of SI microbiota of baseline (gray circles), mock-treated (black squares) and IAV-infected mice (red triangles) pooled from four independent cages are depicted for indicated days of sampling. **b** 2D-PCoA plots of SI microbiota of mock-treated (blue triangle) or IAV-infected mice (red circle) mice at indicated time points post infection. Each symbol represents one individual mouse. Percentages explain variation in PC1 (*x*-axis) and PC2 (*y*-axis). **c** Mean UNIFRAC distances for the comparison of indicated experimental groups. Statistical significance ((*) towards all-within group (#) all between group) was determined by two-tailed student’s *T* test. **d** Relative abundance (%) of indicated bacteria is depicted for individual mock (black symbols) or IAV-infected (red symbols) mice. **e** Normalized abundance of total or indicated groups of bacteria based on qPCR (16S/18S) samples is shown; each symbol represents one animal; mock-treated (black) and IAV-infected (red). Pooled data from four independent cages are depicted (*n* = 18 per time point, per group from two independent experiments, representative data shown). **f** Individual bacterial titers (cfu/organ) and median determined under aerobic (left) and anaerobic conditions (right) on Columbia or Chocolate agar are depicted for mock treated and IAV infected (black for mock, red for H5N1, green for H1N1 (*n* = 6 per group per condition, two independent experiments))

We observed a substantial but transient reduction of community richness in SI microbiota 7 dpi as indicated by a drop in Shannon H-index (Fig. 3a, (*n* = 18 animals per time point and experimental group)). In line with this finding, we observed a separation of over-all microbiota beta diversity from infected and mock-treated animals in PCoA starting from 5 dpi and more pronounced 7 dpi (Fig. 3b, mock *n* = 18, IAV *n* = 16). Comparison of all intestinal samples within the same coordinate system shows a clear shift of beta diversity of intestinal microbiota in mice infected with IAV 7 dpi (Additional file 7: Figure S3B). Analysis of weighed UNIFRAC distances between PCoA group values revealed a significant increase of separation of mock-treated samples vs IAV-infected sample at 7 dpi (Fig. 3c and see Additional file 8: Table S5 for statistics). Interestingly, comparison of mock vs mock distances revealed no increase at this time point, while comparison of IAV vs IAV showed enhanced spreading of beta diversity of intestinal microbiota among infected animals at 7 dpi (Additional file 7: Figure S3C). For later time points, we observed again a reduction of weighed UNIFRAC distances, indicating a transient effect of IAV infection on beta diversity of SI microbiota. Importantly, the shift in beta diversity of SI microbiota between mock-treated and IAV-infected mice 7 dpi occurred independent of cage effects as demonstrated for 7 dpi SI samples (Additional file 7: Figure S3D). In parallel to viral clearance (14 dpi), microbiota from mock-treated and IAV-infected mice were regrouped in the PCoA and stabilized long-term (28 dpi). This implicates a transient effect of acute respiratory infection with IAV on SI microbiota. Next, we analyzed differences in genus OTU abundance that contribute to the observed changes in alpha and beta diversity. SI microbiota from mock-treated animals is composed of S24-7 (71%), *Lactobacillus* (5%), *Sutterella* (3%), *Allobaculum* (2%), and *Clostridiales* (2%). Seven days post infection, we detected a dramatic reduction of *Bacteroidetes* (S24-7: 18%) and an increase of *Firmicutes* (e.g., Lactobacillus 14%) (Additional file 7: Figure S3G, mock *n* = 18, IAV *n* = 16). 16S rRNA gene-specific NGS revealed IAV infection induced substantial reorganization of commensal microbiota composition in the SI 7 dpi, characterized by a drop of *Bacteroidia* and a relative increase of *Gammaproteobacteria* and *Bacilli* (Fig. 3d, mock *n* = 18, IAV *n* = 16). In contrast to LRT microbiota, we saw substantial reduction (> 10×) in overall microbial content in the SI (affecting the two major phyla *Bacteroidetes* and to a lesser extend *Firmicutes*, as shown by specific qPCR 16S/18S (Fig. 3e, *n* = 18 animals, representative data shown). The different extend of depletion of Bacteroidetes and Firmicutes explains the discrepancy between relative 16S rRNA gene sequencing results and the qPCR approach. 18S RNA levels remained constant in all samples, ruling out an impact of total organ mass on the observed reduction of 16S/18S (data not shown). As for the beta diversity, we did not observe an effect of individual cages on Shannon H-index, taxonomic composition, analyzed by 16S rRNA gene NGS (*n* = 18), or 16S/18S levels, measured by qPCR (*n* = 6) for 7 dpi SI samples (Additional file 7: Figure S3E). We confirmed that depletion of gut microbiota is not a specific consequence of H5N1 IAV infection. Pan-16S rRNA gene-specific qPCR of SI samples of mice infected with A/Netherlands/602/2009 (pandemic H1N1, *n* = 5 animals per experimental group) or A/Puerto Rico/8/1934 (H1N1, *n* = 8 animals per experimental group) revealed markedly reduced total bacterial content in SI of infected animals 6 or 7 dpi (Additional file 7: Figure S3F), respectively. We thus concluded that reduction of bacterial content in the SI tract is a general consequence of IAV infection, independent of viral subtype. Accordingly, the number of cultivatable bacteria (limited to anaerobics) was reduced under these conditions (Fig. 3f, *n* = 6, pooled). Of note, the majority of small intestinal bacteria are still difficult to cultivate or non-cultivatable, explaining the quantitative discrepancy between Fig. 3e and f). Surprisingly, the changes in SI microbiota did not directly translate into substantial qualitative or quantitative changes of fecal microbiota (Additional file 9: Figure S4A–E), as indicated by unchanged alpha and beta diversity (Additional file 9: Figure S4B and C) or cultivable bacteria (Additional file 9: Figure S4F). This implicates either a very localized effect of IAV infection on SI microbiota or an overall more robust microbiota in the distal gut than in the proximal small intestine, likely due to generally higher bacterial loads (reviewed in [24]). Since reduction of commensal bacteria and maximum body weight loss coincided, we tested if the reduced uptake of food and water as consequence of typical sickness behavior could cause a reduction of bacteria in the small intestine. Assuming that body weight loss is a direct consequence of starving, we correlated percentage of initial body weight and 16S rRNA gene-specific qPCR results for total bacterial amounts for each animal on 7 dpi. The low *R* score reveals no indication for starving as main driver of microbial content changes in the SI (Additional file 10: Figure S5, mock *n* = 18, IAV *n* = 16). The same holds true for individual phyla changing as consequence of IAV infection (Additional file 10: Figure S5).

Taken together, these data suggest a robust but transient alteration of SI microbiota mostly driven by depletion of total bacterial mass as a consequence of IAV infection. Compositional changes are largely confined to the SI and not extending to the fecal microbiota. This coincided with a surprisingly subtle effect on the respiratory microbiota.

### IAV triggers a systemic type I interferon response but IFN application is not sufficient to provoke small intestine microbiota depletion

**Fig. 4:**
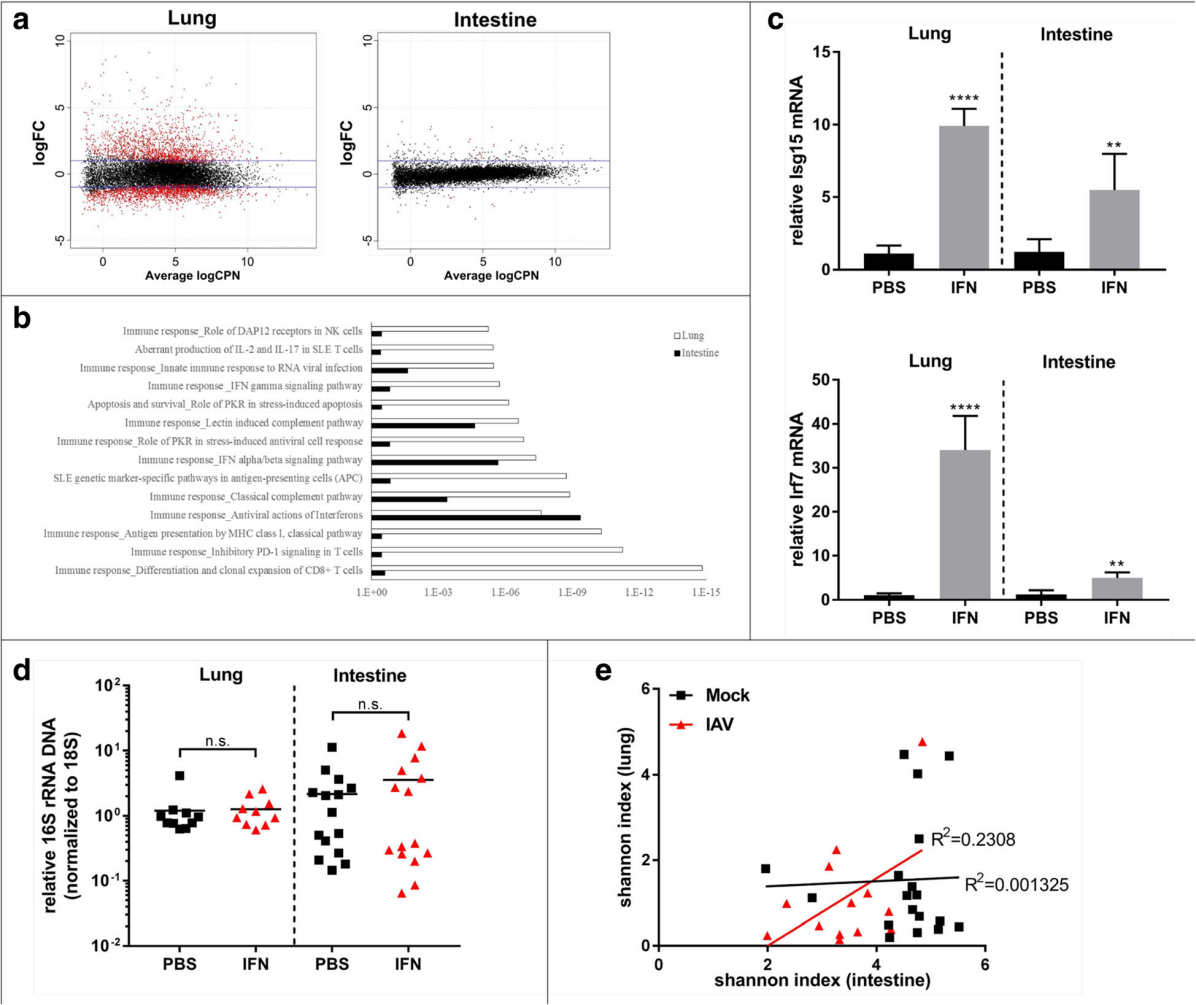
Influenza A virus infection provokes a robust antimicrobial host response in the LRT with less pronounced effects on the small intestine. **a** MA-plots of differentially expressed mRNAs in IAV-infected vs. mock-treated animals. Blue lines indicate twofold threshold; dots represent average n-fold expression values over mock (log2 scale, *p* value < 0.05) of mRNAs from 4 individual IAV-infected mice, 7 days post treatment. RNAseq results from three or four individual mice (mock or IAV, respectively) from lung tissue are depicted on the left, from small intestine on the right. **b** Gene ontology of significantly enriched pathways. White bars represent *p* values for lung-enriched pathways of upregulated mRNAs based on RNAseq results of IAV infected over mock. Black bars represent small intestine-enriched pathways. **c** n-fold induction of ISG15 and IRF7 24 h after IFN-α B/D treatment in lung and small intestine (*n* = 5 per group). **d** n-fold changes in 16S/18S from three independent experiments for SI (*n* = 15 per group) and two independent experiments for LRT (*n* = 10 per group). Median is indicated for each experimental group. **e** Correlation of alpha diversity of lung and intestinal samples for individual mice 7 dpi. Respective *R*^2^ values and regression fit are indicated

To better understand the differences in local (LRT) and distal (SI) host response to IAV infection, we performed global transcriptome analysis of both organs (*n* = 3 for mock, *n* = 4 IAV infected), using RNAseq. As expected and previously published [26], IAV infection mounts a substantial inflammatory and interferon (IFN) driven antimicrobial response in lung tissue 7 dpi (Fig. 4a). Accordingly, histology of lung samples revealed substantial infiltration of monocytic cells into the infected lungs and induction of tissue inflammation (Fig. 1d). In contrast, under the chosen conditions, very few mRNAs are differentially regulated in the small intestine (Fig. 4a, right panel). The majority of upregulated genes are known type I IFN-induced genes, although the level of expression is much lower than observed in the lung. Gene ontology analysis revealed only two functional groups with comparable enrichment in both lung and intestine: antiviral response/type I IFN response and to a lower extend complement system-related genes (Fig. 4b). Since we did not detect viral replication in the small intestine, we wanted to rule out passive transport of viral RNA into the intestinal tissue, which could trigger IFN responses. Neither by qPCR nor by RNAseq we found viral RNA (of + or − sense) in the SI samples 7 dpi, ruling out a direct induction of IFN production in the SI (Additional file 11: Figure S6, (*n* = 5 per experimental group). Accordingly, we did not detect an increase of type I, II, or III IFN mRNA in the SI transcriptome (data not shown), which could implicate that systemic IFN produced in a distant site reaches the small intestine to induce gene expression.

Deriu and colleagues showed reduced impact of IAV infection on fecal microbiota composition in IFNAR^−/−^ mice [22]. Since the receptor is deleted in all tissues, the authors could not rule out indirect effects of a blunted host response in the lung on the intestinal tissue in these animals. Regardless, to test if type I IFN would be sufficient to induce depletion of SI microbiota, we injected recombinant IFNα-B/D systemically into mice and determined total bacterial abundance by 16S/18S qPCR 8 and 24 h post injection. Systemic IFN-α provoked a robust interferon stimulated gene (ISG) response in the SI (Fig. 4c, mock 8 h *n* = 4, IFN-α treated 8 h *n* = 5, mock 24 h *n* = 5, IFN-α treated 24 h *n* = 5) but did not recapitulated the depletion of SI microbiota (Fig. 4d, *n* = 15 animals per experimental group for SI, *n* = 10 animals for lung samples). This implicates a rather indirect effect of type I IFN on intestinal microbiota as suggested previously [22]. In order to understand if LRT and SI microbiota are regulated by the same mechanism, we correlated alpha diversity of microbiota from both organs of the same animal. The *R*^2^ value of 0.23 implicates no common mechanism for microbiota regulation in the lung and small intestine (Fig. 4e, *n* = 18 for mock, *n* = 13 for IAV infected).

Despite having a robust antimicrobial response in the lung, we find little changes in the lung microbiota; conversely, in the small intestine, a weak host response is accompanied by a substantial depletion of microbial mass.

### IAV-induced small intestine microbiota depletion facilitates bacterial pathogen invasion

**Fig. 5:**
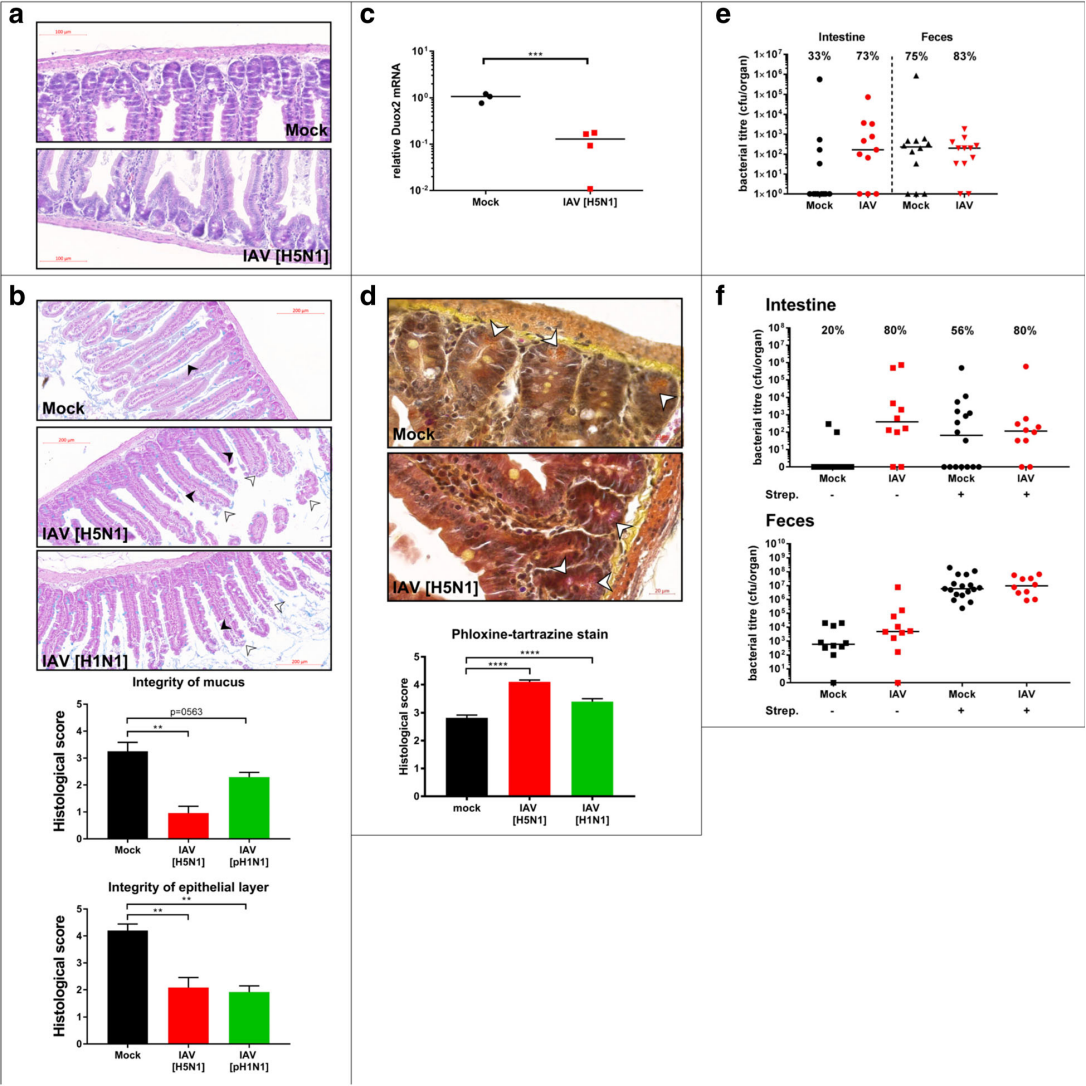
Influenza A virus infection causes small intestinal mucosal tissue damage and activates Paneth cells. **a** Representative H&E stained longitudinal sections of SI are shown (from six independent samples). **b** Representative Alcian Blue staining of longitudinal sections of SI tissue from mock-treated or IAV-infected mice. Tissue damage and mucus layer destruction are indicated by white or black arrows heads, respectively. Quantification of tissue damage and mucus layer integrity (lower panels) by single-blinded scorings of sections from six individual mice (7 dpi or mock treatment, from two independent experiments) performed by two independent researchers are depicted. **c** 18S normalized specific qPCR results (n-fold over mock) for DUOX2 are depicted for individual mice (mock (*n* = 3), IAV (*n* = 4)). **d** Representative pictures of phloxine-tartrazine stained SI sections or mock-treated or IAV-infected mice. Lower panel: quantification of two independent blinded scorings of samples for six mice per experimental group from two independent experiments. **e** Bacterial titers of intracellular *S. typhimurium* in SI tissue. Numbers indicate percentage of invaded samples per experimental group. Individual mice from two independent experiments and median are depicted, SI samples (left side), fecal samples (right side) (mock intestine (*n* = 12) and feces (*n* = 12), iav intestine (*n* = 11), feces (*n* = 12)). **f** Bacterial titers of intracellular *S. typhimurium* in SI tissue. Numbers indicate percentage of invaded samples per experimental group. Individual mice from two independent experiments and median are depicted, SI samples (left side), fecal samples (right side) (*n* = 10 per group except Mock Strep + (*n* = 16 intestine, *n* = 17 feces)

Next, we wanted to assess if IAV infection alters SI tissue architecture under the chosen infection conditions. H&E staining of intestinal sections (*n* = 6 per experimental group, representative data shown) revealed pronounced epithelial shedding and destruction of villi, but no signs of inflammatory infiltrates (Fig. 5a). Chronic dysbiosis has been associated with a reduction in mucosal thickness in inflammatory bowel disease (summarized in [27]). To evaluate if mucosal thickness in the intestinal tract is affected by IAV infection, we stained SI sections of mock-treated and IAV-infected animals with Alcian blue (Fig. 5b). IAV infection results in robust elimination of the mucosal layer in the small intestine. Interestingly, Goblet cell staining is unchanged, implicating either a defect in mucus layer maintenance or a reduction in mucus secretion. To monitor if this reduction of microbial content in the SI provokes a transcriptional host response, we looked for expression of Duox2 mRNA as an intestinal marker gene, which is reduced in the intestine of antibiotic treated and germ-free animals [28]. In line with these findings, Doux2 mRNA levels are reduced in intestinal samples of mice infected with IAV (Fig. 5c, *n* = 3 mock, *n* = 4 IAV infected). To address a potential mechanism of bacterial depletion, we stained SI sections with phloxine-tartrazine for acidophilic granules in Paneth cells. These granules contain antimicrobial peptides, which were shown to be important for maintenance of microbial homeostasis (summarized in [29]). SI sections from IAV-infected mice show significantly more intense staining of Paneth cell granules (Fig. 5d, *n* = 6 animals per experimental group) without changes in overall Paneth cell numbers per crypt (data not shown).

A major function of commensal microbiota is the occupation of ecological niches in different body sites providing direct competition to invading pathogenic bacteria. In fact, pretreatment of mice with a single oral gavage of streptomycin efficiently removes intestinal microbiota, thus allowing successful infection of otherwise fairly resistant animals with gastrointestinal pathogens such as *S. typhimurium* [4]. We thus hypothesized that a reduction of SI commensals and mucus layer would leave certain niches unoccupied and the host vulnerable for bacterial super infection.

We tested if IAV-mediated reduction of bacteria in the SI sensitize the host for *S. typhimurium* attachment and invasion by infecting 10 dpi, when mice recovered from virus-induced weight loss. We treated SI organ samples with gentamycin to exclude *S. typhimurium* in the lumen of the intestine from the analysis. Inoculation with *S. typhimurium* resulted in robust epithelial invasion in about 70% of IAV pre-infected animals (Fig. 5e, left side *n* = 12 for mock, *n* = 11 for IAV infected), while only one third of mock treated animals showed invasion of *S. typhimurium* into SI epithelium. Since we did not observe a significant difference in intracellular bacterial titers between those mock-treated and IAV-infected animals that show super infection, we proposed that the commensal microbiota pose a threshold, thus reducing the chance for colonization of the SI with *S. typhimurium*, without limiting the intracellular replication, once this threshold is overcome. IAV infection does not appear to improve overall growth conditions for *S. typhimurium* in the intestine since we did not observe an enhancement of *S. typhimurium* titers after IAV infection in fecal pellets (Fig. 5e, right side, *n* = 12 per group), which is in accordance with the rather mild effects we observed on fecal microbiota. These data implicate a direct competition mechanism for SI microbiota against attachment of invading bacterial pathogens to intestinal epithelium, which can be hampered temporarily by IAV-induced dysbiosis. Phylum-specific qPCR revealed that streptomycin treatment like IAV infection has a profound effect on *Bacteroidetes* in the SI but little impact on abundance of *Firmicutes* (Additional file 12: Figure S7), which suggests that *Bacteroidetes* are important for opposing *S. typhimurium* invasion. To causally link reduction of SI microbiota and enhanced bacterial super infection after IAV infection, we first decided to eliminate gut microbiota by treatment of mock- or IAV-infected animals with streptomycin and super infect these animals with *S. typhimurium*. The extent of super infection with *S. typhimurium* did not increase when IAV infection and streptomycin treatment were combined, ruling out microbiota-independent effects of enhanced *S. typhimurium* invasion (Fig. 5f, *n* = 10 animals per experimental group (for mock with streptomycin treatment *n* = 17).

Our data show that depletion of microbiota as a consequence of IAV infection increases susceptibility to enteric bacterial infections, in line with previous studies showing enhanced sensitivity of germ-free, gnotobiotic, or antibiotic-treated animals to *S. typhimurium* infection [4, 30].

## Discussion

Increased sensitivity to super infection of the respiratory tract following IAV infection has been known for decades from clinical cases and mouse models (summarized in [31]). However, mechanisms for bacterial super infection of the lung are still debated and likely involve factors of virus [32, 33], host [31, 34–38], and potentially local bacterial microbiota [19], albeit for the latter a formal proof of causality is still missing. This recent study correlated IFN type III-signaling with IAV-mediated changes in host microbiota, increased bacterial loads in the URT, and *S. aureus* URT super infection [19]. Our infection experiments do not result in significant changes of microbiota abundance of URT or LRT. This could be due to the uses of different virus strains or the method of sample collection (lavage [19] vs. total organ homogenate in our study). However, by culturing lung homogenates on rich agar media, we observed significant outgrowth of *Enterobacteriaceae* in IAV-infected mouse LRT. In dysbiotic conditions, it is consequently feasible that these bacteria find more favorable growth conditions supporting the hypothesis that IAV infection lays ground for outgrowth of preexisting pathobionts [20] or invading pathogenic bacteria in the respiratory tract. If microbiota competition is indeed involved in this process, our data implicate that a change in composition rather than a change in overall bacterial abundance in the respiratory tract is responsible for the reduced threshold towards bacterial invasion after IAV infection. Most strikingly, infection with IAV resulted here in reduction of commensal community richness and substantial quantitative depletion of small intestinal commensals. It is currently unclear, if changes in microbiota composition of lung and intestine are induced by a similar mechanism. Correlation of alpha diversity 7 dpi revealed no convincing indication of this, but it should be pointed out that stability and dynamics of microbial communities in these two organs are substantially different. Future studies will have to address the underlying local and systemic, regulatory circuits. Reduced community richness or reduced overall abundance of the intestinal microflora was previously described to increase attachment and invasion of enteric pathogens [4, 30]. Recently, reduction of microbial diversity in gut microbiota was observed in H7N9-infected patients [23], which is in line with our findings and could explain the subsequently elevated sensitivity to *S. typhimurium* invasion. Since additional treatment with streptomycin does not enhance the super infection caused by IAV, we conclude the sensitivity is directly linked to absence of competition by commensal microbiota and not through an indirect mechanism. Interestingly, both streptomycin treatment and IAV infection preferentially deplete *Bacteroidetes* (mainly S24-7) in the SI. It is thus possible that this poorly characterized group of bacteria poses a major barrier to *S. typhimurium* super invasion. Our data also implicate that probiotics treatment could potentially balance the IAV-induced dysbiosis. Type I interferon was implicated in regulating microbial contents in fecal pellets in response to IAV infection [22]. It should however be pointed out that the mice used in this study lack IFNAR systemically, thus indirect effects cannot be ruled out. This is of importance since the authors mainly show phenotypic differences in context of combined IAV and *S. typhimurium* infection, but not with single IAV infection. Here, we show that IFN-α application is not sufficient to recapitulate the effect of IAV on SI microbiota abundance. However, we cannot rule out that efficient microbiota changes require longer exposure times or different types of IFN. Regardless, depletion of microbiota would require directly acting antibacterial effector molecules. It currently remains unclear what is the exact nature of these effector molecules, but the increased staining of Paneth cell granules implies a potential involvement of antimicrobial peptides in IAV-mediated depletion of SI microbiota.

## Conclusions

To our best knowledge, this is the first study addressing microbiota kinetics after acute viral infection in the LRT and the SI. To our surprise, IAV infection mediates bacterial super infection in the SI tract by quantitative depletion of bacterial content, while in the respiratory tract subtle, qualitative changes in microbiota appear to change growth conditions and reduce the threshold against invading pathogenic bacteria. The transient nature of the IAV-induced dysbiosis in both lung and intestine underlines the stability of microbial communities in both sampling sites, but also reveals the temporary vulnerability in response to inflammatory events. Our study further underlines the importance of gathering qualitative, as well as quantitative data when studying microbiota kinetics. We currently cannot exclude that repetitive infections or underlying conditions of the host organism could favor permanent imprinting of microbial communities by acute infections.

## Methods

### Mice, housing conditions, infection, IFN treatment, sampling, and temperature measurement

C57BL/6J mice (female, 8–9 weeks of age) were purchased from Charles River Laboratories (France) and housed under SPF/BSL2 conditions. Animals were treated in two independent cages per experiment in two independent repeats (4 cages in total per experimental group) in groups of 3–6 animals/cage. All animals were housed for 7 days to adjust to housing conditions under a strict 12-h light/dark cycle and fed *ad libitum*. For IAV and *S. pneumoniae* infection, mice were injected i.p. a mix of ketamin/xylazine (100 and 5 mg/kg, respectively) in 100 μl of sterile PBS. Upon reaching deep anesthesia, mice were inoculated with 40 μl of PBS or virus or bacterial suspension via the intranasal route, respectively. Streptomycin (200 μg/mouse) was applied by oral gavage 24 h prior to *S. typhimurium* infection. *S. typhimurium* was introduced by oral gavage of 200 μl of bacterial suspension in sterile PBS using a blunt-end steel feeding needle. Recombinant human hybrid IFN-α B/D [39] was kindly provided by Dr. P. Stäheli (University of Freiburg, Germany). It was applied by i.p. injection of 6000 U in 200 μl of sterile PBS. Body weights were measured daily during the light phase. Body temperature was measured using a contact-free thermal camera (FLIR E60, FLIR Thermography, USA). Fresh fecal pellets were collected during the weighing procedure. Upon reaching experimental or humane endpoints (85% of initial body weight), animals were euthanized using controlled CO_2_ exposure. Organs were sampled immediately after euthanasia using sterile tools. Tools were changed in between organs and experimental groups to avoid cross-contamination. Organ and fecal samples were immediately stored at − 70 °C until extraction of DNA.

### DNA extraction

Microbiota composition alters substantially within one organ due to local differences in O_2_ and CO_2_ concentration, pH, and other environmental and host factors [12, 40–42]. To assure the majority of microbes of a given sample site (including cell- or biofilm-associated bacteria [43]) were captured, we decided to extract DNA from total organ homogenates. Total lung (from lower trachea downwards) and the proximal 5 cm of small intestine (covering distal duodenum and proximal jejunum) were collected. For lung and small intestine, DNA extraction was performed using Ultra Clean Tissue & Cells DNA isolation kit (MoBio, USA) according to manufacturer’s protocol with slight modifications. Briefly, whole organ samples were homogenized with ¼′′ stainless steel grinding balls (MPBio, USA) in 1 ml homogenization buffer, using a Bead Blaster 24 (Benchmark Scientific, USA) with a speed setting of 6 m/s for 90s and 30s break, repeated 6 times. One hundred microliters of total organ homogenate were used for further DNA extraction steps following the kits’ guidelines. DNA from fecal samples was extracted with PowerLyzer PowerSoil DNA isolation kit (MoBio, QIAGEN, USA) according to manufacturer’s instructions with homogenization setting described above. DNA was quantified using a Nanodrop ND 1000 spectrophotometer (Thermo Scientific, USA). DNA samples were normalized to a concentration of 50 ng/μl.

### Library construction and Illumina sequencing

The V4 region of 16S rRNA gene was targeted for PCR amplification using modified universal bacterial 16S primer pair (515F/806R) [44]. The forward primer, 5’-AATGATACGGCGACCACCGAGATCTACAC-TATGGTAATT-GT-GTGCCAGCMGCCGCGGTAA-3′, consists of an Illumina Adapter, a forward primer pad, forward primer link, 515F, and the reverse primer, 5’-CAAGCAGAAGACGGCATACGAGAT-NNNNNNNNNNNN-AGTCAGTCAG-CC-GGACT ACHVGGGTWTCTAAT-3′, includes the reverse complement of an Illumina 3′ adapter, Golay barcode (12 bp indicated by N), reverse primer pad, reverse primer linker, and 806R, given in respective order above. For each sample, PCR reactions were carried out in triplicates of 25 μl reaction volume, each time using 1 μl of template DNA (50 ng), 1 μl of each modified 515F (5 μM) and 806R (5 μM), 12 μl molecular biology grade water (Amimed, BioConcept, Switzerland), and 10 μl 5PRIME Hot Master Mix (QuantaBio, USA). Thermal cycling consist of an initial denaturation step at 94 °C for 3 min, followed by 35 cycles of denaturation at 94 °C for 45 s, annealing at 50 °C for 60 s and extension at 72 °C for 90 s, with a final extension at 72 °C for 10 min. Triplicates of each amplicon were pooled, visualized on a 2% agarose gel, and quantified with Quant-iT Picogreen dsDNA Assay kit (Life Technologies, USA) according to manufacturer’s protocol using Gemini EM Microplate Reader (Molecular Devices, USA). One hundred nanograms of amplicon for each sample were pooled into a library and cleaned using QIAquick PCR purification kit (QIAGEN, USA). Libraries were quantified by Qubit fluorimeter (Life Technologies) using Qubit dsDNA BR Assay Kit (Invitrogen, USA). Eighteen picometers of each library were mixed with PhiX DNA (10%) and were loaded on a MiSeq Reagent kit V2 (500 cycles) together with customized sequencing primers; read1, 5’-TATGGTAATTGTGTGCCAGCMGCCGCGGTAA-3′, read2, 5’-AGTCAGTCAGCCGGACTACHVGGGTWTCTAAT-3′ and index read, 5′- ATTAGAWACCCBDGTAGTCCGGCTGACTGACT-3′. 250 bp paired-end sequencing was performed on the MiSeq platform (Illumina, USA) in the iGE3, Institute of Genetics and Genomics in Geneva, CMU, University of Geneva. Sequencing results were obtained and de-multiplexed using standard method supplied by the MiSeq, Illumina platform.

### Control for environmental contamination

As negative controls (blanks), we used empty sample tubes from the respective DNA isolation kit: blanks for organ samples (blanks A): Ultra Clean Tissue & Cells DNA isolation kit (MoBio, USA), blanks for fecal pellets (blanks B): PowerLyzer PowerSoil DNA isolation kit (MoBio, QIAGEN, USA). These empty sample tubes underwent the whole respective extraction procedure (including all respective buffers used) without contact to mouse material. Blank libraries were constructed together with organ libraries on the same 96-well plate, to measure potential cross contamination during PCR. DNA concentrations for blank libraries were below the limit of detection by picoGreen. Thus, for the pooled libraries, we used the maximum volume, defined by the lowest concentrated organ DNA or fecal DNA sample of the corresponding pooled library, respectively.

### Bioinformatics pipeline

Sequence analysis performed with the open source software package QIIME following a pipeline described previously [45] with slight modifications. Briefly, forward and reverse reads were joined with a minimum 200 bp overlap between two reads using default joining method, fastq-join. Unpaired reads were excluded from downstream analysis. Barcodes were extracted using standard QIIME script, and remaining reads were formed into a single library with additional sequence quality filtering (Phred score of 20) [46]. Chimeric reads were detected and filtered using UCHIME [47], and Operational Taxonomic Units (OTUs) and their respective taxonomy were assigned to sequences in the library following a closed reference OTU picking strategy taking Greengenes (v.13.8) core reference database [48] as a reference with a 97% sequence identity match as a cutoff [49, 50]. The OTU table was further filtered of OTUs having less than 0.1% abundance within each biological sample. OTUs found in the blanks for organ samples are listed in Additional file 1 Table S1.

Within-community diversity (α-diversity), degree of differentiation among biological samples (β-diversity) and relative abundance of bacterial communities were calculated using standard QIIME workflow (core_diversity_analysis.py) with sampling depth of 200, 400, 20,000 OTUs, chosen by a PyCogent algorithm, for the lung, the small intestine, and fecal samples, respectively. Chao1 estimator [51, 52] and Shannon H-index [53] were used to evaluate community richness through alpha diversity (Additional files 2, 3, 7*,* and 9: Figures S1a, S2a, S3a, and S4a). For phylogenic beta diversity, distance matrices were formed using weighted UniFrac [54] and Principle Coordinate Analysis (PCoA) based on all samples of each sampling site and visualized through 2D PCoA plots using Qiime. Taxa for each OTU table were summarized via QIIME visual representation, or graphs were produced based on calculated abundance tables using GraphPad Prism v7.

### RNA extraction

Organs were collected 7 days post mock treatment or infection, respectively, and stored in RNAlater (Qiagen) overnight, then stored at − 70 °C until further use. Total RNA was extracted using TRIzol (Invitrogen), following manufacturer’s instruction. Briefly, total organs were thawed and taken out from RNAlater and homogenized three times with ¼′′ stainless steel grinding balls (MPBio, USA) in 1 ml TRIzol solution using a Bead Blaster 24 (Benchmark Scientific, USA) with a speed setting of 6 m/s for 30″ and 30″ break on ice. Organ homogenates were kept at room temperature for 10′ to allow efficient lysis of the tissue. 0.2 ml chloroform was added to each sample and incubated for another 10′ at room temperature. Samples were centrifuged for 15′ at 12000 × *g*. The upper aqueous phase was carefully transferred into a new tube and mixed with 0.5 ml isopropanol and incubated for 10′ at room temperature. RNA was precipitated by centrifugation at 12000 × *g* for 10′ at 4 °C, followed by a wash using 75% ethanol and re-precipitation at 8000 × *g* for 5′. Pellets were air-dried and re-suspended in 100 μl RNase-DNase free water. All steps, including centrifugations, during isolation were performed on ice or at 4 °C, respectively, using nuclease-free reagents and plastic.

### qPCR

For 16S rRNA gene DNA quantification, 1 μl of DNA extracts from organ homogenates was mixed with 10 μl of 2X KAPA SYBR FAST qPCR Master Mix-Universal (KAPABiosystems, USA), 0.4 μl of each Forward (10 μM) and Reverse (10 μM) primer, and 8.2 μl of RNAse, DNase Free Molecular Biology Grade Water (Amimed, BioConcept, Switzerland). Quantitative PCR was performed following a thermal cycling protocol of an initial denaturation step at 95 °C for 10 min, followed by 45 cycles of denaturation at 95 °C for 15 s, annealing/extension at 61.5 °C for 60 s, with a final melting curve step from 60 to 95 °C with 0.5 °C increment.

Panbacteria, *Bacteroidetes*, and *Firmicutes* specific primers were described previously [55]. Levels were normalized to 18S rRNA gene DNA values (18Sfw: 5’-GTAACCCGTTGAACCCCATT-3′ and 18Srev: 5’-CCATCCAATCGGTAGTAGCG-3′), [55]. For RNA levels where stated, 1 μg total RNA was used to synthesize cDNA using SuperScript II Reverse Transcriptase kit following kit’s guidelines (Invitrogen, USA). Quantitative PCR was performed using 2X KAPA SYBR FAST qPCR Master Mix-universal according to manufacturer’s instructions. These specific primers for murine cDNAs were used: mIsG15fw: 5’-ATGAACGCTACACACTGCATC-3′, mISGrev5’-CCATCCTTTTGCCAGTTCCTC-3’ mIrf7fw: 5’-GAGACTGGCTATTGGGGGAG-3′, mIrf7rev: 5’-GACCGAAATGCTTCCAGGG-3′, mDuox2fw 5’-ACGCAGCTCTGTGTCAAAGGT-3′, mDuox2rev: 5’-TGATGAACGAGACTCGACAGC-3′.

### RNAseq

Total RNA was quantified with a Qubit fluorimeter (Life Technologies) and RNA integrity assessed with a Bioanalyzer (Agilent Technologies). The TruSeq mRNA stranded kit from Illumina was used for the library preparation with 800 ng of total RNA as input. Library molarity and quality was assessed with the Qubit and Tapestation using a DNA high sensitivity chip (Agilent Technologies). A pool of 22 libraries was loaded at 8.5 pM for clustering on three lanes of a Single-read Illumina Flow cell. Reads of 50 bases were generated using the TruSeq SBS HS v3 chemistry on an Illumina HiSeq 2500 sequencer. Quality control (QC) was done with FastQC v.0.11.5. Reads were mapped with TopHat2 v.2.0.11. Biological QC was done with PicardTools v.1.141. Reads per gene feature were counted with HTSeq v.0.6p1. Normalization was performed according to the design model with R/Bioconductor edgeR v. 3.4.2. Gene ontology analysis was performed using significantly upregulated mRNAs (IAV vs. mock, cutoff twofold, *p* < 0.05) as input for Metacore (GeneGo, Thomson Reuters) pathway analysis tools.

### Data availability

16S rRNA gene NGS data were deposited at NCBI Bioproject (https://www.ncbi.nlm.nih.gov/bioproject/) under the accession numbers PRJNA419860 for URT samples, PRJNA419861 for SI samples, PRJNA419862 for fecal pellet samples, and PRJNA419895 for blank controls. RNAseq data are deposited to NCBI GEO (https://www.ncbi.nlm.nih.gov/geo/) under the accession number GSE107488**.**

### Virus

Reverse genetics systems were kindly provided by Dr. Peter Palese and Dr. Adolfo García-Sastre (Icahn School of Medicine at Mount Sinai, New York, NY, USA). Viruses were rescued as previously described [56]. IAV A/Viet Nam/1203/2004 (VN/1203) HALo (low pathogenic version), A/Puerto Rico/8/1934 (PR/8), and A/Netherlands/602/2009 (Neth/602) were described previously [15, 57–59]. Virus stocks were grown at 37 °C for 48 h in 11-day-old, embryonated chicken eggs (VN/1203 and PR/8) purchased from the Animalerie d’Arare, University of Geneva or on Madin Darby Canine Kidney cells (MDCK, ATCC)(Neth/602). Viruses were plaque purified on MDCK, and genomic RNA was extracted for sequence confirmation. Virus titers of serially diluted organ homogenates or virus stocks were determined by standard plaque assay [60]. Virus stocks were stored at − 70 °C. All procedures involving infectious virus were performed strictly under BSL-2 conditions in accordance with federal guidelines.

### Bacteria

Cultivatable URT, LRT and SI bacteria, were grown from serially diluted organ homogenates on Chocolate Agar (Polyvitex) or Columbia agar + 5% sheep blood plates (Biomerieux, France) under aerobic or unaerobic conditions. Anaerobic conditions were achieved by Genbag anaerobic bag in GenBox with supplied indicator (Biomerieux, France). Colony forming units (cfu) were determined after 24 h. The naturally streptomycin resistant *S. typhimurium* strain SL1344 was kindly provided by Dr. WD Hardt (ETH Zurich) and grown in LB + 100 μg/ml streptomycin. Overnight cultures were diluted 1:300 and grown for additional 3 h 45 min to reach ~ 10e9 cfu/ml. Bacteria were pelleted at 3500 × g for 10 min, washed, and resuspended in PBS in a final concentration of estimated 5 × 10e8 cfu/ml. Colony forming units of inoculum was determined by serial dilution and plating on LBagar + Strep. Tissue invaded *S. typhimurium* was quantified by a modified gentamicin protection assay [61]. Briefly, SI tissue was rinsed with ice cold PBS and incubated with 50 μg/ml gentamicin solution for 30 min. Residual gentamicin was removed by washing with ice cold PBS. Tissue was homogenized as described above, and serial dilutions of homogenate were spotted in triplicates onto pre-dried LB-plates containing 200 μg/ml streptomycin. CFU were determined 24 h later. *S. pneumoniae* (ATCC-6303) was purchased from LGC (Germany) and cultivated on Trypticase soy agar plates with 5% sheep blood (Biomerieux, France). Liquid cultures were grown according to ATCC instructions, to an OD_600_ of 0.5 in Trypticase soy broth (Biomerieux, France). Mice were infected with 1500 cfu via the intranasal route as described above.

### Colony PCR for identification of bacterial colonies

To identify specific colonies grown from lung homogenates plated on Columbia agar + 5% sheep blood (Biomerieux, France), 16S rRNA gene was targeted for PCR amplification using Taq polymerase kit (ThermoFisher, USA) with two sets of primers pairs; 515F/806R [44] and 8Fhomd, 5’-GAGTTTGATCMTGGCTCAG-3′, 1510Rhomd, 5′- TACCTTGTTACGACTT-3′ (kindly provided by Dr. Vladimir Lazarevic, University of Geneva), according to manufacturer’s instructions. Briefly, colonies were picked from designated plates and diluted in 100 μl molecular biology grade water (Amimed, BioConcept, Switzerland). 10 μl of each homogenate was mixed with 5 μl 10X Taq buffer, 1 μl dNTP mix (10 mM), 1 μl of each forward primer (5 μM) and reverse (5 μM), 5 μl MgCl_2_ (2.5 mM), and 0.25 μl Taq polymerase (5 U/μl) and filled up to 50 μl with molecular biology grade water. PCR mixes were subjected to thermal cycling consisting of an initial denaturation step of 95 °C for 10 min, followed by 35 cycles of denaturation at 95 °C for 30 s, annealing at 60 °C for 30 s, and extension at 72 °C for 60 s, with a final extension at 72 °C for 10 min. Following PCR reactions, samples were separated on a 1% agarose gel, and corresponding PCR product was cut out using a scalpel. DNAs from cut agarose bits were recovered using ZymoClean Gel DNA recovery kit (USA) following manufacturer’s protocol. Recovered DNA samples were sent to Sanger Sequencing (Microsynth, Switzerland) with corresponding forward primer used for PCR amplification. Obtained sequences were aligned to matching OTU sequence stated in Greengenes database (OTU ID 1111294, 1107027) using CLC Sequence Viewer (QIAGEN, USA). Sequences were also blasted against 16S rRNA gene database on NCBI Blast website.

### Histological scoring

Organs were fixed in 4% formaldehyde, embedded in paraffin and cut. Slices were stained with Alician Blue for mucopolysaccharides and counterstained with nuclear red (Sigma Aldrich) [62]. A second set of slices was stained with hematoxylin and eosin [62]. Paneth cell staining was performed as described previously using phloxine tartrazine staining [62]. Scoring of intestinal epithelial shedding, mucus integrity and Paneth cell granula staining, was performed single-blinded by two independent researchers using an arbitrary scale from 0 to 5.

### Statistics

In order to determine statistical significance, we applied unpaired *t* tests to body weight loss data, body temperature data, and RT-qPCR data using Graph Pad Prism 7.0. Bacterial titers from mouse organs, Shannon indices, OTU abundance, and histology scoring results were analyzed with Mann-Whitney test; ****p ≤ 0.0001, ***p ≤ 0.001, **p ≤ 0.01, *p ≤ 0.05, *ns* not significant.

## Additional files

Additional file 1: Table S1. Overview of animal number used per experiment and indication of organ samples that did not pass quality control for bioinformatics analysis. (XLSX 11 kb)

Additional file 2: Figure S1. A) rarefection plots for baseline samples of LRT, SI, feces, and blanks: number of sequences is plotted against Chao1 estimator for each sample. (B) 2D PCoA plots of untreated mice (baseline), lung (green), intestine (orange), feces (blue), blank A & B (red). All samples were taken from the same respective animals. (C) Mean relative OTU abundance of baseline samples (LRT, SI, Feces, and Blanks A&B). (PDF 1340 kb)

Additional file 3: Figure S2. (A) rarefection plot: number of sequences is plotted against Chao1 estimator. Error bars indicate SE. (B) Most abundant OTU found in baseline, mock or IAV-infected LRT samples 7 dpi on genus level. Median relative abundance (%) is depicted. (C) N-fold changes of 16S/18S DNA levels normalized to mean of mock samples of each day examined. Individual mice and median are depicted (*n* = 18 per group per time point, two independent experiments, representative data shown). (D) Individual lung titers of mock treated (black circles) and IAV (pH1N1, Neth/602) infected mice (red squares), median titers (pfu/organ) are indicated. Limit of detection (LoD) 50 pfu (*n* = 5 per group). (E) Relative abundance of total bacteria and *Firmicutes* based on qPCR (16S/18S) normalized to mean of mock samples is shown for individual mice and indicated time points treated with mock (black symbols) or indicated viruses (red symbols for pH1N1 6 dpi) (*n* = 5 per group). (F) Bacterial titers of cultivatable URT microbiota 7 dpi grown on indicated agar plates (*n* = 6 per group). (G) Sequence alignment of Sanger sequencing results of 16S rRNA gene V4 region of *Enterobacteriaceae* (OTU ID 1111294) colonies. (H) Sequence alignment of Sanger sequencing results of 16S rRNA gene V4 region of *Lactobacillaceae* (OTU ID 1107027) colonies. (PDF 1691 kb)

Additional file 4: Table S2. Read counts for individual organ, fecal, and blank samples. Statistics for each group are indicated in the upper part of the table. (XLSX 10 kb)

Additional file 5: Table S3. Relative taxonomic composition of blank samples for kit A and kit B for individual samples (%) (XLSX 15 kb)

Additional file 6: Table S4. Relative abundance (%) of OTU 1111294 in indicated sample sites for baseline, mock, and IAV-infected animals. (XLSX 12 kb)

Additional file 7: Figure S3. (A) rarefection plot: number of sequences is plotted against Chao1 estimator. Error bars indicate SE. (B) 2D PCoA of SI microbiota mock-treated (blue symbols) or IAV-infected mice (red symbols) at indicated time points post infection. (C) Mean UNIFRAC distances for the comparison of indicated experimental groups. Statistical significance was determined by two-tailed student’s *T* test. (D) 2D PCoA of SI microbiota mock-treated (blue symbols) or IAV-infected mice (red symbols) at indicated time points post infection. Each symbol represents one mouse. Each symbol type refers to an individual cage. **(E)** Shannon index, relative abundances of indicated bacterial classes determined by 16S rRNA gene NGS, and relative 16S/18S levels of animals 7 dpi are shown. Each symbol represents one animal. Each symbol type refers to an individual cage. (F) Relative abundance of total bacteria based on qPCR (16S/18S) normalized to median of mock samples is shown for individual mice and indicated time points treated with mock (black symbols) or indicated viruses (red symbols) for for H1N1 7 dpi (*n* = 8 per group) pH1N1 6 dpi (*n* = 5 per group). (G) Most abundant OTU on genus level. Median relative abundance (%) is depicted. (PDF 1956 kb)

Additional file 8: Table S5. Statistical testing of distances in beta diversity for indicated pairings (XLSX 12 kb)

Additional file 9: Figure S4. (A) Rarefection plot: number of sequences is plotted against Chao1 estimator. Error bars indicate SE. (B) Alpha diversity depicted as shannon index for fecal microbiota of baseline (gray), mock-treated (black) or IAV-infected (red) mice. Individual values per mouse and median are depicted. (C) 2D PCoA plots of fecal microbiota mock-treated (blue) or IAV-infected mice (red) at indicated time points post infection. Each symbol represents one mouse. (D) Most abundant OTU on genus level. Median relative abundance (%) is depicted. (E) Relative abundance (%) of significantly changing OTUs of individual mice and median are depicted (mock red, IAV black). (F) Cultivatable bacterial cfu/fecal pellet of mock-treated (black) or IAV-infected (H5N1 red, H1N1 green) 7 dpi grown under aerobic or anaerobic conditions are depicted (*n* = 6 per group per time point, two independent experiments). (PDF 1343 kb)

Additional file 10: Figure S5. correlation of total bacteria or individual bacterial groups with body weight loss for individual animals (mock-treated (black), IAV-infected (red)) for small intestinal microbiota. Respective *R*^2^ values and regression fit are indicated. (PDF 914 kb)

Additional file 11: Figure S6. mean copy number of minus strand (vRNA) and plus strand (mRNA/cRNA) RNA copies ± SD per organ sample are depicted as determined by specific RT-qPCR; *n.d.* not detected. (PDF 215 kb)

Additional file 12: Figure S7. phylum-specific qPCR for streptomycin vs. mock-treated mice. Individual relative levels of Bacteroidetes or Firmicutes normalized to 18S and median are depicted. Pooled data from two independent mouse experiments are shown (*n* = 5 per group). (PDF 717 kb)

## Abbreviations

bp: Base pair
BSL: Biosafety level
cfu: Colony forming unit
COPD: Chronic obstructive pulmonary disease
dNTP: Deoxy nucleotide triphosphate
dpi: Days post infection
H&E: Haematoxylin and eosin
HAlo: Hemagglutinin low pathogenic
IAV: Influenza A virus
IBD: Inflammatory bowl disease
IFN: Interferon
IFNAR: Interferon alpha receptor
LoD: Limit of detection
LRT: Lower respiratory tract
MDCK: Madin Darby canine kidney cells
NGS: Next generation sequencing
OTU: Operational taxonomic unit
PC: Principle coordinates
PCoA: Principle coordinates analysis
PCR: Polymerase chain reaction
pfu: Plaque forming units
qPCR: Quantitative polymerase chain reaction
rRNA: Ribosomal RNA
SI: Small intestine
SPF: Specific pathogen free
U: Unit
URT: Upper respiratory tract
