# Supplementary figures and images for: Influenza A virus infection impacts systemic microbiota dynamics and causes quantitative enteric dysbiosis
Source: Microbiome. 2018 Jan 10;6:9. doi: 10.1186/s40168-017-0386-z (PMC5763955; doi:10.1186/s40168-017-0386-z)

Figure S1

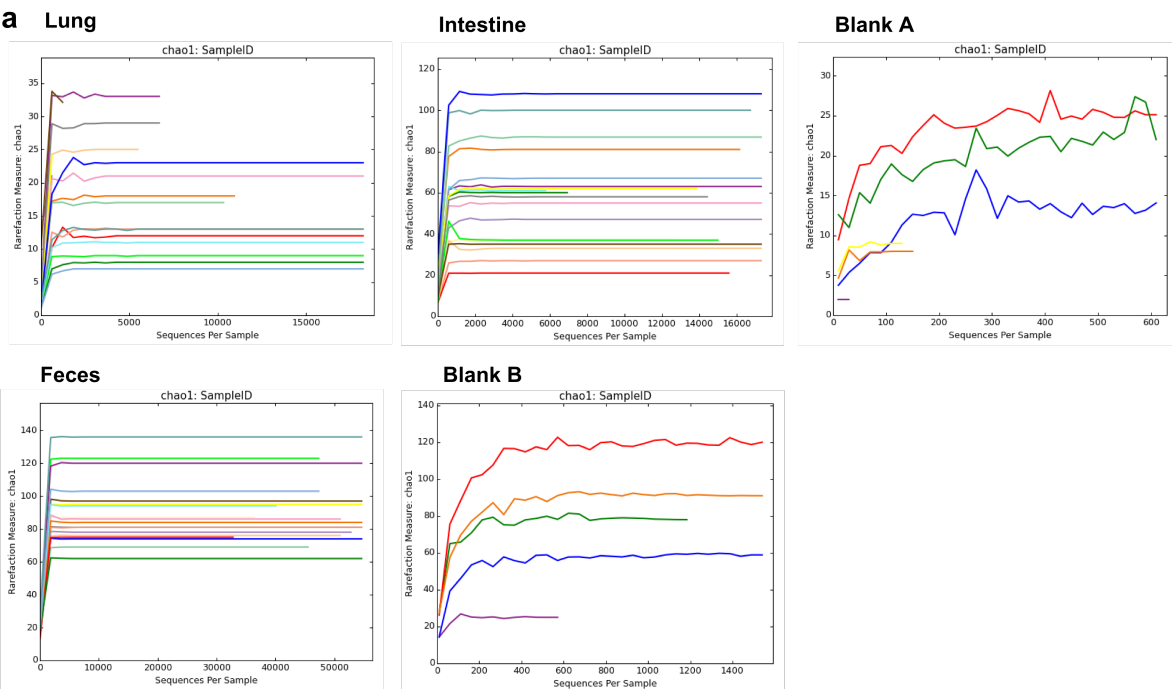

b

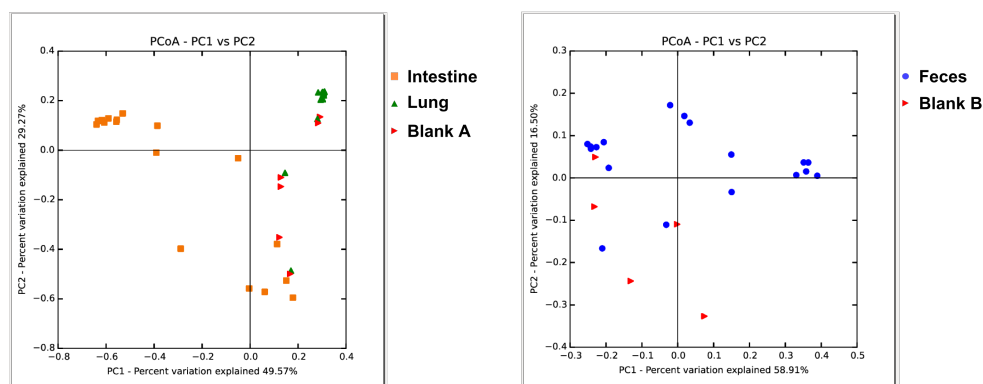

c

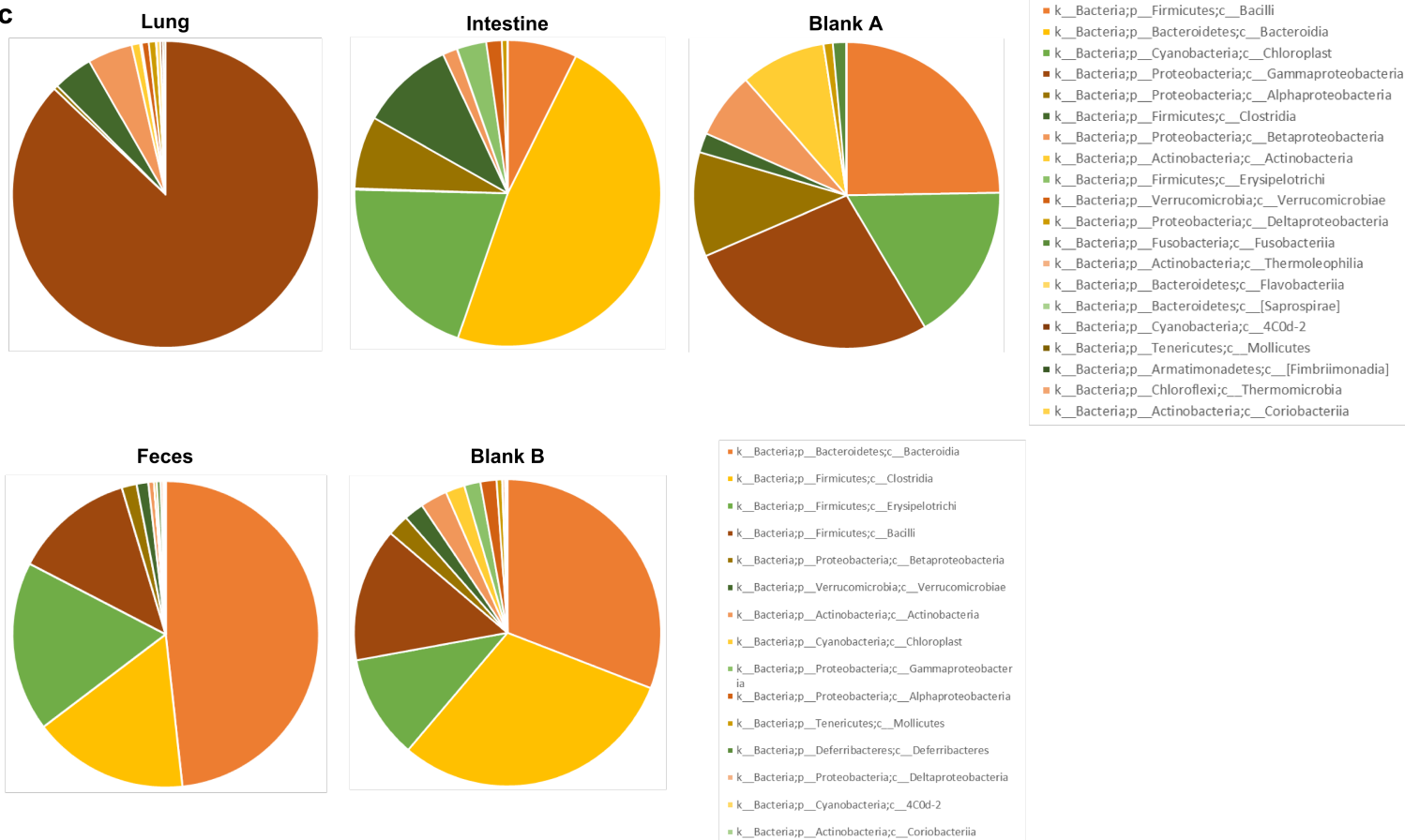

Supplement: Supplementary file 2 — A) rarefection plots for baseline samples of LRT, SI, feces, and blanks: number of sequences is plotted against Chao1 estimator for each sample. (B) 2D PCoA plots of untreated mice (baseline), lung (green), intestine (orange), feces (blue), blank A & B (red). All samples were taken from the same respective animals. (C) Mean relative OTU abundance of baseline samples (LRT, SI, Feces, and Blanks A&B). (PDF 1340 kb) [file 40168_2017_386_MOESM2_ESM.pdf]

Figure S2

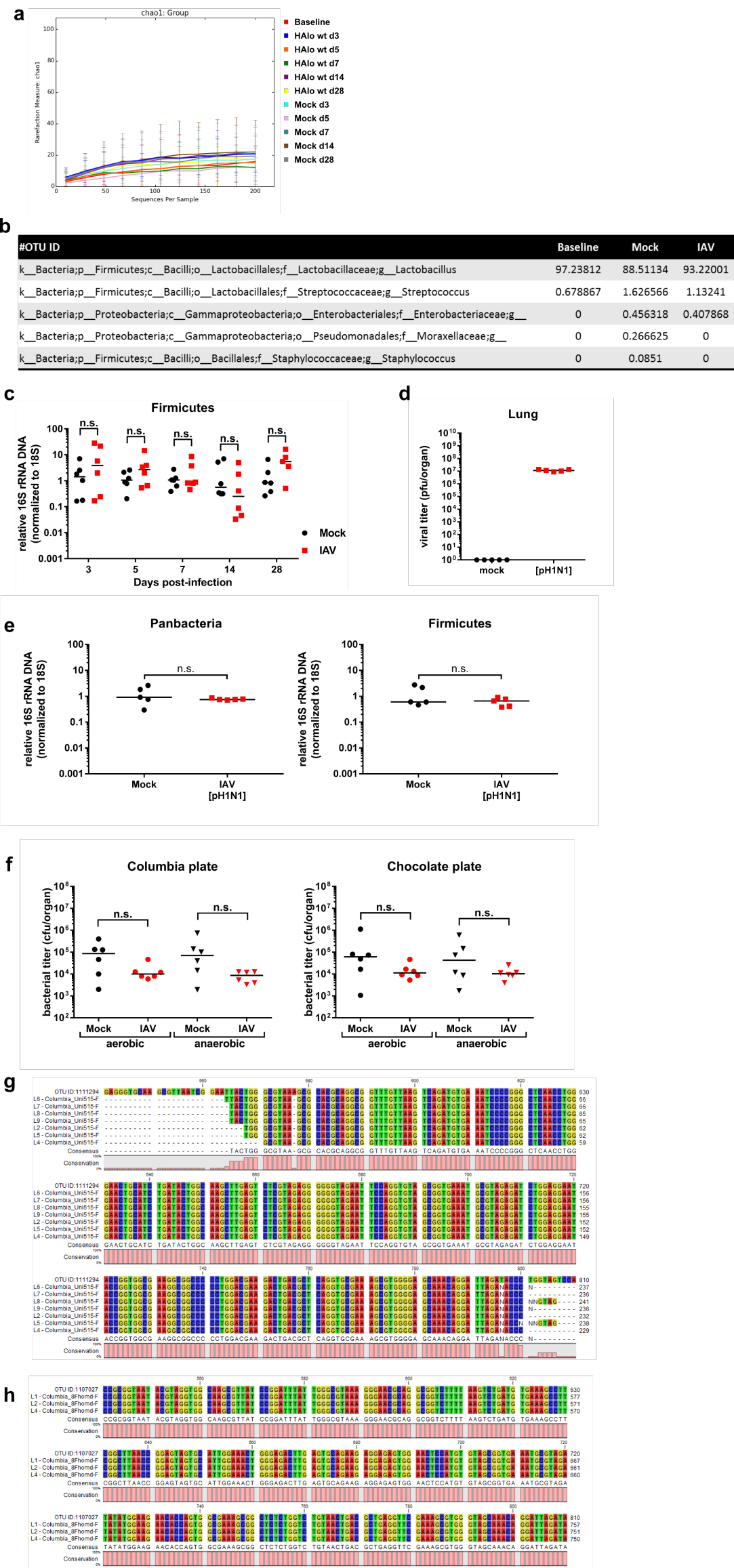

Supplement: Supplementary file 3 — (A) rarefection plot: number of sequences is plotted against Chao1 estimator. Error bars indicate SE. (B) Most abundant OTU found in baseline, mock or IAV-infected LRT samples 7 dpi on genus level. Median relative abundance (%) is depicted. (C) N-fold changes of 16S/18S DNA levels normalized to mean of mock samples of each day examined. Individual mice and median are depicted (n = 18 per group per time point, two independent experiments, representative data shown). (D) Individual lung titers of mock treated (black circles) and IAV (pH1N1, Neth/602) infected mice (red squares), median titers (pfu/organ) are indicated. Limit of detection (LoD) 50 pfu (n = 5 per group). (E) Relative abundance of total bacteria and Firmicutes based on qPCR (16S/18S) normalized to mean of mock samples is shown for individual mice and indicated time points treated with mock (black symbols) or indicated viruses (red symbols for pH1N1 6 dpi) (n = 5 per group). (F) Bacterial titers of cultivatable URT microbiota 7 dpi grown on indicated agar plates (n = 6 per group). (G) Sequence alignment of Sanger sequencing results of 16S rRNA gene V4 region of Enterobacteriaceae (OTU ID 1111294) colonies. (H) Sequence alignment of Sanger sequencing results of 16S rRNA gene V4 region of Lactobacillaceae (OTU ID 1107027) colonies. (PDF 1691 kb) [file 40168_2017_386_MOESM3_ESM.pdf]

Figure S3

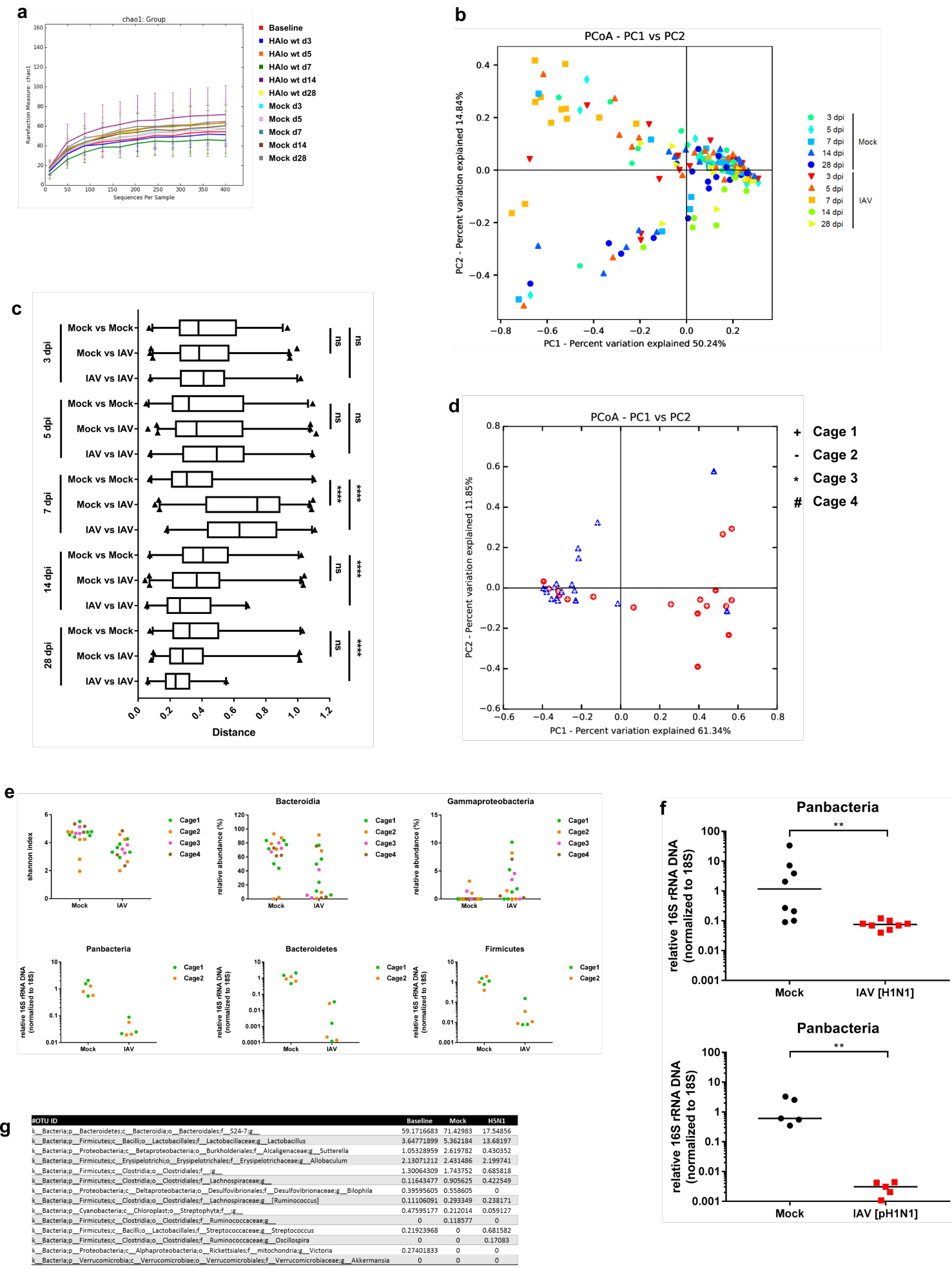

Supplement: Supplementary file 7 — (A) rarefection plot: number of sequences is plotted against Chao1 estimator. Error bars indicate SE. (B) 2D PCoA of SI microbiota mock-treated (blue symbols) or IAV-infected mice (red symbols) at indicated time points post infection. (C) Mean UNIFRAC distances for the comparison of indicated experimental groups. Statistical significance was determined by two-tailed student’s T test. (D) 2D PCoA of SI microbiota mock-treated (blue symbols) or IAV-infected mice (red symbols) at indicated time points post infection. Each symbol represents one mouse. Each symbol type refers to an individual cage. (E) Shannon index, relative abundances of indicated bacterial classes determined by 16S rRNA gene NGS, and relative 16S/18S levels of animals 7 dpi are shown. Each symbol represents one animal. Each symbol type refers to an individual cage. (F) Relative abundance of total bacteria based on qPCR (16S/18S) normalized to median of mock samples is shown for individual mice and indicated time points treated with mock (black symbols) or indicated viruses (red symbols) for for H1N1 7 dpi (n = 8 per group) pH1N1 6 dpi (n = 5 per group). (G) Most abundant OTU on genus level. Median relative abundance (%) is depicted. (PDF 1956 kb) [file 40168_2017_386_MOESM7_ESM.pdf]

**Figure S5**

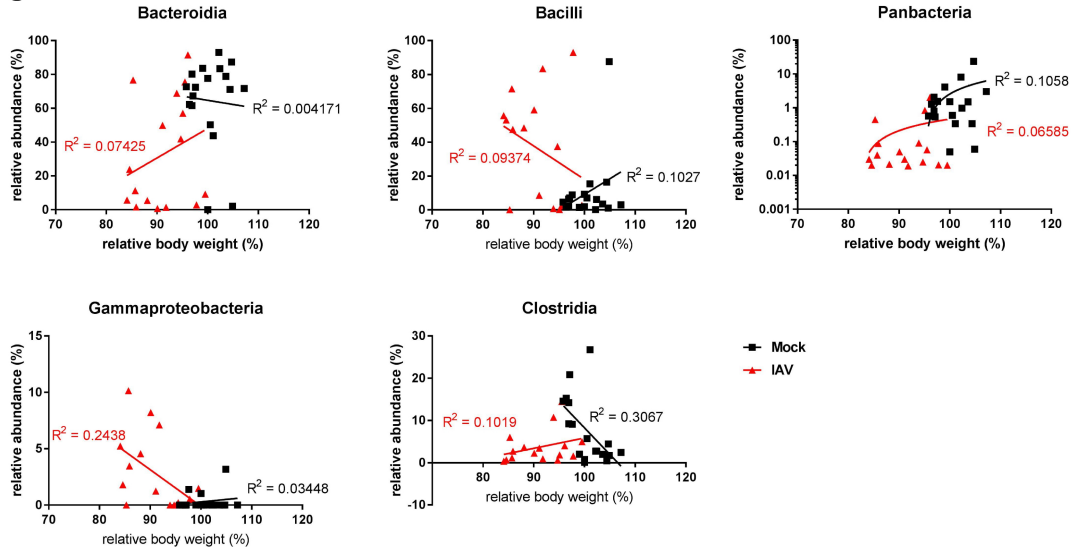

Supplement: Supplementary file 10 — correlation of total bacteria or individual bacterial groups with body weight loss for individual animals (mock-treated (black), IAV-infected (red)) for small intestinal microbiota. Respective R2 values and regression fit are indicated. (PDF 914 kb) [file 40168_2017_386_MOESM10_ESM.pdf]

# Figure S6

**a**

**vRNA**

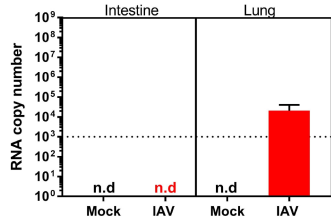

**b**

**mRNA**

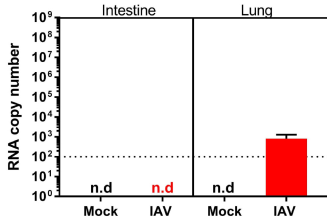

Supplement: Supplementary file 11 — mean copy number of minus strand (vRNA) and plus strand (mRNA/cRNA) RNA copies ± SD per organ sample are depicted as determined by specific RT-qPCR; n.d. not detected. (PDF 215 kb) [file 40168_2017_386_MOESM11_ESM.pdf]

**Figure S7**

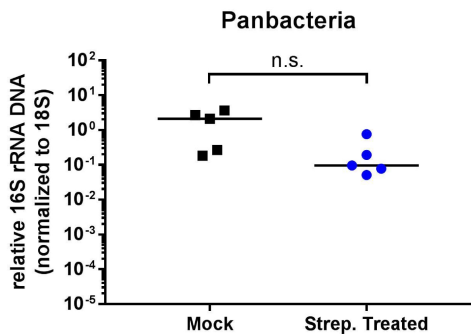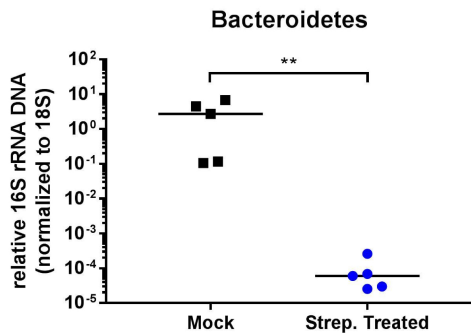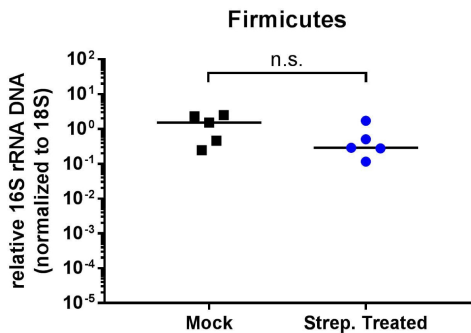

Supplement: Supplementary file 12 — phylum-specific qPCR for streptomycin vs. mock-treated mice. Individual relative levels of Bacteroidetes or Firmicutes normalized to 18S and median are depicted. Pooled data from two independent mouse experiments are shown (n = 5 per group). (PDF 717 kb) [file 40168_2017_386_MOESM12_ESM.pdf]
