# Supplementary material for: Influenza A virus infection impacts systemic microbiota dynamics and causes quantitative enteric dysbiosis
Source: Microbiome. 2018 Jan 10;6:9. doi: 10.1186/s40168-017-0386-z (PMC5763955; doi:10.1186/s40168-017-0386-z)

Figure S4

a

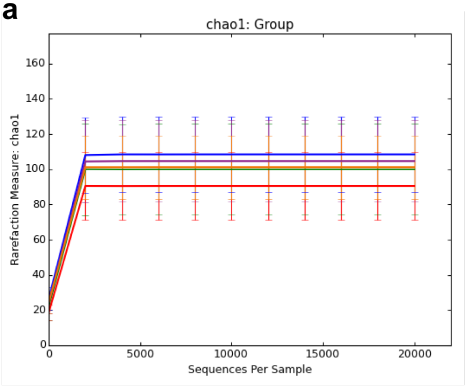

b

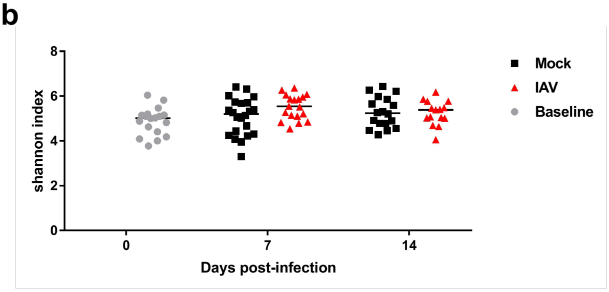

c

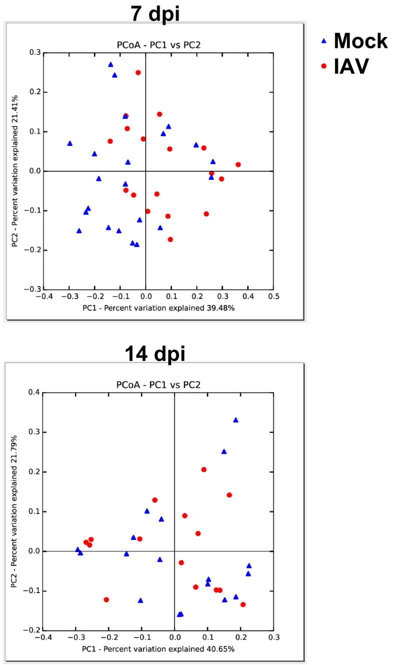

d

#OTU ID	Baseline	Mock	HALo
k__Bacteria;p__Bacteroidetes;c__Bacteroidia;o__Bacteroidales;f__S24-7;g__	37.6202527	28.24089	28.81687
k__Bacteria;p__Firmicutes;c__Erysipelotrichi;o__Erysipelotrichales;f__Erysipelotrichaceae;g__Allobaculum	15.8569435	21.53645	8.852242
k__Bacteria;p__Firmicutes;c__Bacilli;o__Lactobacillales;f__Lactobacillaceae;g__Lactobacillus	11.5225025	5.195663	3.856821
k__Bacteria;p__Firmicutes;c__Clostridia;o__Clostridiales;f__;g__	7.36071427	14.78678	17.93602
k__Bacteria;p__Bacteroidetes;c__Bacteroidia;o__Bacteroidales;f__Rikenellaceae;g__	4.31237215	3.16604	5.324386
k__Bacteria;p__Bacteroidetes;c__Bacteroidia;o__Bacteroidales;f__Bacteroidaceae;g__Bacteroides	3.06879659	3.002717	5.485645
k__Bacteria;p__Proteobacteria;c__Betaproteobacteria;o__Burkholderiales;f__Alcaligenaceae;g__Sutterella	1.62233742	1.576213	1.051216
k__Bacteria;p__Firmicutes;c__Clostridia;o__Clostridiales;f__Lachnospiraceae;g__	1.52274066	1.320677	2.357211
k__Bacteria;p__Bacteroidetes;c__Bacteroidia;o__Bacteroidales;f__Prevotellaceae;g__Prevotella	1.07025467	1.058659	1.839902
k__Bacteria;p__Firmicutes;c__Clostridia;o__Clostridiales;f__Ruminococcaceae;g__	0.68997921	1.117355	2.281283
k__Bacteria;p__Bacteroidetes;c__Bacteroidia;o__Bacteroidales;f__;g__	0.48450671	0.389377	0.26805
k__Bacteria;p__Firmicutes;c__Clostridia;o__Clostridiales;f__Lachnospiraceae;g__[Ruminococcus]	0.45259461	0.366296	0.733849
k__Bacteria;p__Firmicutes;c__Clostridia;o__Clostridiales;f__Ruminococcaceae;g__Oscillospira	0.18854669	0.956433	1.622378
k__Bacteria;p__Verrucomicrobia;c__Verrucomicrobiae;o__Verrucomicrobiales;f__Verrucomicrobiaceae;g__Akkermansia	0.17569801	0.221608	0.941342
k__Bacteria;p__Proteobacteria;c__Deltaproteobacteria;o__Desulfovibrionales;f__Desulfovibrionaceae;g__Bilophila	0.16398189	0.169266	0.521753
k__Bacteria;p__Actinobacteria;c__Actinobacteria;o__Bifidobacteriales;f__Bifidobacteriaceae;g__Bifidobacterium	0.08116738	0.715412	0.507451
k__Bacteria;p__Tenericutes;c__Mollicutes;o__RF39;f__;g__	0.06591157	0	0.189328
k__Bacteria;p__Tenericutes;c__Mollicutes;o__Anaeroplasmatales;f__Anaeroplasmataceae;g__Anaeroplasma	0.06506324	0	0
k__Bacteria;p__Bacteroidetes;c__Bacteroidia;o__Bacteroidales;f__Porphyromonadaceae;g__Parabacteroides	0.05649505	0	0.274587
k__Bacteria;p__Firmicutes;c__Clostridia;o__Clostridiales;f__Ruminococcaceae;g__Ruminococcus	0.05637174	0.116903	1.178755
k__Bacteria;p__Cyanobacteria;c__4C0d-2;o__YS2;f__;g__	0	0.063537	0.208045
k__Bacteria;p__Firmicutes;c__Clostridia;o__Clostridiales;f__Lachnospiraceae;g__Coprococcus	0	0	0.617868
k__Bacteria;p__Deferribacteres;c__Deferribacteres;o__Deferribacterales;f__Deferribacteraceae;g__Mucispirillum	0	0	0.499442
k__Bacteria;p__Firmicutes;c__Erysipelotrichi;o__Erysipelotrichales;f__Erysipelotrichaceae;g__	0	0	0.061256

e

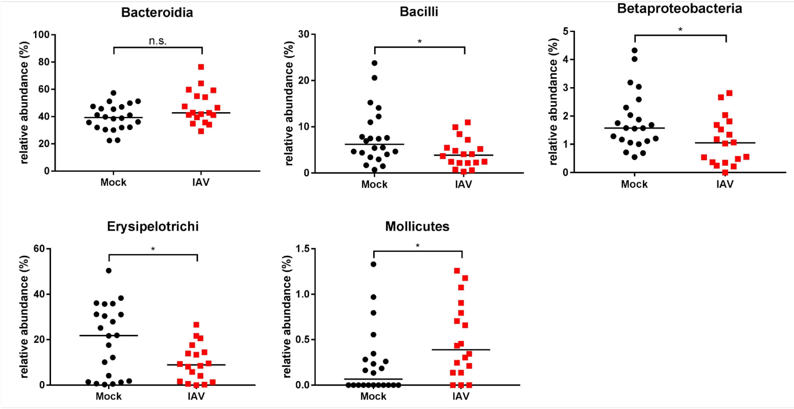

f

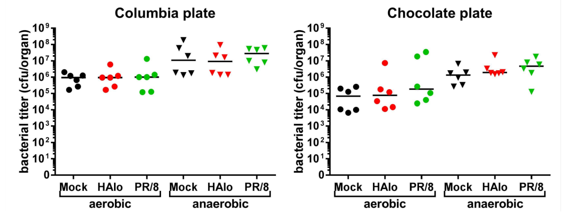

Supplement: Supplementary file 9 — (A) Rarefection plot: number of sequences is plotted against Chao1 estimator. Error bars indicate SE. (B) Alpha diversity depicted as shannon index for fecal microbiota of baseline (gray), mock-treated (black) or IAV-infected (red) mice. Individual values per mouse and median are depicted. (C) 2D PCoA plots of fecal microbiota mock-treated (blue) or IAV-infected mice (red) at indicated time points post infection. Each symbol represents one mouse. (D) Most abundant OTU on genus level. Median relative abundance (%) is depicted. (E) Relative abundance (%) of significantly changing OTUs of individual mice and median are depicted (mock red, IAV black). (F) Cultivatable bacterial cfu/fecal pellet of mock-treated (black) or IAV-infected (H5N1 red, H1N1 green) 7 dpi grown under aerobic or anaerobic conditions are depicted (n = 6 per group per time point, two independent experiments). (PDF 1343 kb) [file 40168_2017_386_MOESM9_ESM.pdf]
